# Polydimethylsiloxane-Based Composites with Photo-Autocatalytic Properties: Surface Photooxidation, Hydrophobicity, and Nanomechanical Properties

**DOI:** 10.3390/polym18111334

**Published:** 2026-05-28

**Authors:** Mihaela Iuliana Avadanei, Mirela-Fernanda Zaltariov, Iuliana Stoica, Cristian-Dragos Varganici, Diana Elena Ciolacu, Iuliana Spiridon, Adrian Fifere, Ovidiu Gabriel Avadanei

**Affiliations:** 1 “Petru Poni” Institute of Macromolecular Chemistry, 41A Gr. Ghica Voda Alley, 700487 Iasi, Romania; zaltariov.mirela@icmpp.ro (M.-F.Z.); stoica_iuliana@icmpp.ro (I.S.); varganici.cristian@icmpp.ro (C.-D.V.); dciolacu@icmpp.ro (D.E.C.); spiridon@icmpp.ro (I.S.); 2 Centre of Advanced Research in Bionanoconjugates and Biopolymers, “Petru Poni” Institute of Macromolecular Chemistry, 700487 Iasi, Romania; fifere@icmpp.ro; 3 Faculty of Physics, Al. I. Cuza University, 11 Carol I Blvd, 700506 Iasi, Romania

**Keywords:** photoaging, lanthanum complexes, silicon elastomers, Schiff base ligands, photocatalysis

## Abstract

A synergistic approach to the photodegradation of polydimethylsiloxane-based composites upon photoaging was implemented by using La(III) complexes of Schiff base ligands with a silicon-containing spacer as fillers. The analysis methods were spectral, nanomechanical, and morphological. The results show that the accelerated oxidative degradation of the polydimethylsiloxane matrix is due to the combined actions of radicals, fragments, and photoproducts derived from the photolysis of the La(III) complexes and the water vapors in the photoaging chamber. Compared to the undoped polydimethylsiloxane, the photo-excited radical intermediates and photoproducts of the La(III) complexes, with aromatic or quinone structures, in ground or in excited state, have acted as photocatalysts and as new sources for reactive intermediates and for the generation of reactive oxygen species. Infrared, electron spin resonance, and nanomechanical investigations revealed that the chemistry of the photoaged surfaces comprises oxygen–containing species, photoreaction products, and an extended siloxane network with embedded ligand fragments. The key role of La(III) complexes in promoting the generation of reactive species is described. The study highlights the unexplored potential of La(III) complexes of Schiff base ligands bearing a silane/siloxane spacer as potential catalysts in the photodegradation of polymers and plastics.

## 1. Introduction

Polydimethylsiloxane (PDMS) is known for its high UV, chemical, and thermal stability, warranting the sustainability of PDMS-based products and technologies [1]. Converting PDMS from an inert elastomer into a responsive, functional matrix requires breaking this chemical monotony. By altering its surface chemistry or incorporating active fillers, PDMS can be engineered to sense, adapt, conduct, or interact dynamically with its environment [1]. Such hybrid materials combine the structural flexibility, light weight, excellent formability, stretchability, and ease of processing of PDMS with the localized charge density, variable oxidation states, optical d-d or f-f transitions, magnetic properties, and catalytic activity of metal complexes. In particular, integrating photosensitive organometallic compounds into PDMS enables the creation of materials capable of optical sensing [2,3], localized photocatalysis [4,5], light-driven actuation [6,7], or direct laser writing of conductive pathways [7,8]. On the other side, these active fillers alter how PDMS interacts with photons and impact its degradation mechanism. Depending on the material design, this phenomenon is engineered either as a destructive vulnerability (degradation of the composite itself) [9,10] or as an environmental utility (using the composite to photodegrade external pollutants) [4,5,11,12].

Integrating rare-earth elements into polymer composites yields advanced functional materials with highly specific electronic, magnetic, and optical signatures. As not all Ln(III) complexes are capable of energy transfer to achieve the highly desired emissive properties, the non-emitters and particularly La(III) are essential in sustainable energy technologies such as energy storage and fuel cells and in industrial catalytic cracking processes. The known sensitivity of La(III)-based organometallic structures to UV-Vis light enabled them to function as catalysts or photocatalysts of degradation, depolymerization, or cleavage of polymers or contaminants [13,14] and as photosensitizers in photodynamic therapy [15,16].

In this context, the specificity of La(III)-based materials as depolymerizing agents and precursors in organolanthanide chemistry prompted us to explore the catalytic potential of two lanthanum complexes of Schiff base ligands with a silicon-containing spacer for the photoinitiated degradation of PDMS. The special design of the fillers was intended to: (i) overcome the challenge of the interfacial compatibility and to avoid obtaining cloudy, non-homogeneous materials with compromised mechanical integrity; (ii) serve as light absorbers and local triggers of photochemical transformations; (iii) serve as vehicles for introducing rare earth metal ions into the matrix while suppressing their agglomeration. With this approach, we obtained a photoresponsive and multifunctional PDMS composite, and we aimed to investigate its potential for photo-autocatalysis under weathering conditions.

The PDMS-based composites were fabricated as thin films, and we investigated their response to simulated sunlight in a photoaging chamber compared with that of an undoped PDMS film, over 600 h. We started from a series of premises: (i) the slow photodegradation of PDMS in a simulated terrestrial medium without catalysts or additives [17] and the known photochemistry [18,19]; (ii) the catalytic role of La(III) complexes in depolymerization in mild conditions of polycarbonates and polyesters [20,21,22], Nylon-6 [23] or PVC [24]; (iii) the redox activity of the La(III) cation itself, preventing the recombination of photogenerated electrons and holes, regulating therefore the concentration of active •O_2_^−^ and •OH radicals [25,26]; (iv) the influence of the coordinated NO_3_^−^ anions on the course of photoreactions [27]; (v) the catalytic role of the relative humidity in the photoaging chamber in depolymerization, generating •OH radicals, or as a limiting factor in certain reactions [28] and (vi) the presence of reactive oxygen species (ROS, meaning molecular oxygen, OH•, H_2_O_2_, ^1^O_2_, and O_2_^•−^) and of environmental persistent free radicals (EPFRs), well-known as being initiators of degradation [29,30]. Some ROS are based on an energy or electron transfer from the excited host to molecular oxygen or water molecules (the superoxide radical (O_2_^•−^) or the ^1^O_2_ molecule), while others are small fragments eliminated from the host itself or from the environmental water molecules (OH• or OH^−^) [29,30].

We hypothesized that La(III) complexes embedded in PDMS would alter the matrix’s photochemistry and would profoundly affect the properties of the samples’ surface. Analysis of the surface chemical changes was performed by spectral and morphological methods (optical and nanomechanical). The thermal analysis complements the surface investigations by adding information about the bulk structure.

Our research was aimed at determining: (1) the extent of the surface modification as influenced by the La(III) complexes; (2) the role of the La(III) complexes in the photochemistry of the PDMS-based composites; and (3) the degree of hydrophobic recovery and reconstruction of the surface, actions that are in direct relationship with the depth of surface degradation. The changes in chemical structure and in certain properties across samples at different time points during photoaging are finally discussed following the proposed photoaging mechanism for composites. The study presents an original approach to applying lanthanum complexes of ligands with a silicon-containing spacer to photoage PDMS, which shares a common base structure. The findings of this study highlight the untapped potential of non-perovskite, non-emitting lanthanide complexes as initiators of photoreactions and possible photocatalysts.

## 2. Materials and Methods

### 2.1. Materials

Low molecular mass polydimethylsiloxane-*α,ω*-diol (PDMS), M_n_ = 28,000 g mol^−1^, was prepared according to the established procedure [31]. Tetraethyl orthosilicate (TEOS), reagent grade 98%, Dibutyltin dilaurate (DBTL) 95%, Lanthanum(III) nitrate hexahydrate, La(NO_3_)_3_·6H_2_O 99%, 2-Hydroxy-3-methoxybenzaldehyde (*o*-vanillin), 99% were purchased from Sigma-Aldrich (St. Louis, MO, USA). Chloroform (pKa = 15.7) and methanol (pK_a_ = 15.5), HPLC grade, 99.9% LiChrosolv, were achieved from Fisher Scientific (Pittsburgh, PA, USA). The precursors, 1,1,3,3-(tetramethyldisiloxane-1,3-diyl)-bis-(methylene)-bis-(*p*-aminobenzoate) and dimethylsilane-bis-(methylene)-bis-(*p*-aminobenzoate), were prepared as previously reported [32,33]. Acetonitrile (Aldrich, St. Louis, MO, USA), pK_a_ = 25 at 25 °C.

### 2.2. Synthesis of Ligands and of La(III) Complexes

**Scheme 1:**
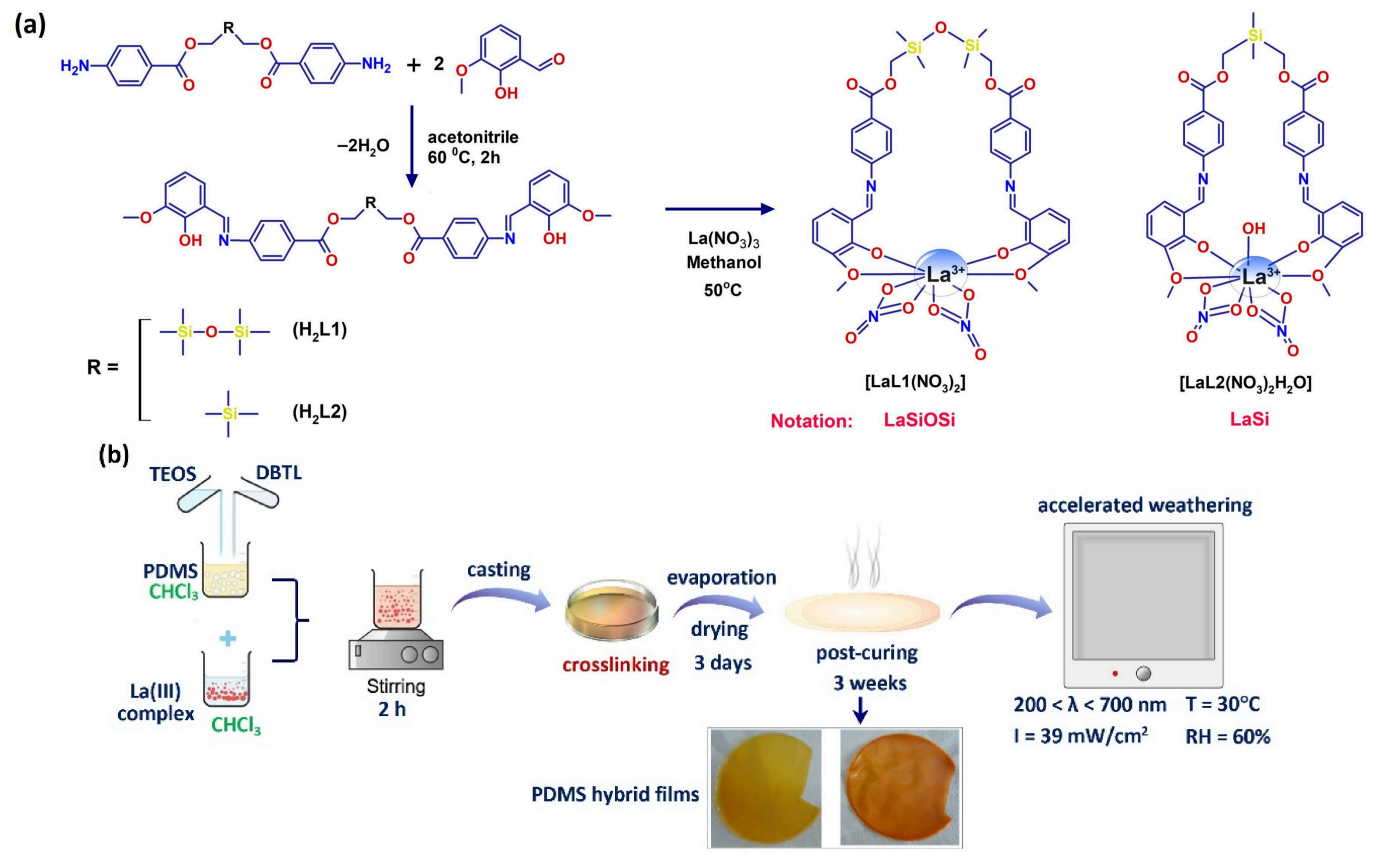
Preparation of PDMS hybrid films: (**a**) Synthetic methodology for obtaining ligands and La(III) complexes; (**b**) Fabrication of PDMS nanocomposite films.

The La(III) complexes were synthesized in two steps, following the procedure described in refs. [34,35] (Scheme 1): (1) the preparation of the Schiff base ligands, (E)-(tetramethyl-disiloxanediyl)-bis(methylene)-bis(4-((E)-(2-hydroxy-3-methoxybenzilidene)-amino)benzoate (H_2_**L1**) and (E)-(dimethylsilane-diyl)-bis(methyl-ene)-bis(4-((E)-(2-hydroxy-3-methoxybenzilidene)amino)-benzoate (H_2_**L2**). The procedure started from 1,1,3,3-(tetramethyldisiloxane-1,3-diyl)-bis-(methylene)-bis-(*p*-aminobenzoate) (0.216 g, 0.5 mmol) or dimetilsilan-bis-(metilen)-bis-(*p*-aminobenzoate) (0.179 g, 0.5 mmol) and o-vanillin (0.152 g, 1 mmol) dissolved in acetonitrile (10 mL, 1:1, v:v) and stirred to reflux for 2 h, followed by filtration, washing, and drying of the precipitate at RT. (2) The preparation of the La(III) complexes continued with mixing the Schiff base products that had been isolated in the first step (0.14 g, 0.2 mmol) and dissolved in methanol (10 mL) with La(III) nitrate (0.065 g, 0.2 mmol) in methanol (5 mL). The resulting mixture was stirred at 50 °C overnight. The precipitate formed was filtered off, washed with cold methanol, and dried at RT. The structural analyses, including FTIR, NMR, and MALDI-MS spectra of ligands and La(III) complexes, are presented in the Supporting Information as Figures S1–S12. The MALDI-TOF/MS analyses confirmed the structure of complexes as being La**L1**(NO_3_)_2_ and La**L2**(NO_3_)_2_ × H_2_O, respectively. The short notation for the two complexes is LaSiOSi for La**L1**(NO_3_)_2_, and LaSi for La**L2**(NO_3_)_2_ × H_2_O.

### 2.3. Fabrication of Hybrid Materials Based on La(III) Complexes

The hybrid films were prepared by the incorporation of 0.025 g La(III) complexes solubilized in CHCl_3_ (10 mL) in a mixture of PDMS (1 g), TEOS (0.5 mL) and catalyst (DBTL, 14 μL) in CHCl_3_ (10 mL) stirred at RT for 30 min. After adding the complex solution, the resulting mixture was stirred at RT for another 2 h, transferred into a Teflon Petri dish and left for evaporation and crosslinking at RT. The hybrid films were removed after 3 days and post-cured for a further 2–3 weeks at RT (Figure S13). The crosslinking matrix is formed by the hydrolysis process of TEOS to Si-OH groups and their condensation with PDMS *α,ω*-OH groups [36]. The lanthanum content in the films is 3830 ppm La(III) for LaSi_PDMS and 3610 ppm La(III) for LaSiOSi_PDMS.

### 2.4. Accelerated Weathering

PDMS-based composites were aged in a laboratory chamber (Angelantoni Ind., Cimacolle PG, Italy). The samples were exposed to artificial light from a mercury lamp (200 < λ < 700 nm, incident light intensity of 39 mWcm^−2^) at a temperature of 30 °C and a relative humidity (RH) of 60%. Periodically, the samples were removed from the exposure chamber at set times (100, 200, 300, 400, 500, and 600 h) and analyzed. The non-irradiated samples were used as references.

### 2.5. Materials Characterization

Elemental analysis. Elemental analysis was performed on an Elemental Exeter Analytical CE 440 apparatus (Exeter Analytical Ltd., Coventry, UK).

Nuclear Magnetic Resonance spectroscopy. The NMR experiments were conducted on a Bruker Avance NEO 400 MHz Spectrometer (Bruker BioSpin GmbH, Ettlingen, Germany) equipped with a 5 mm QNP direct detection z−gradients probe. The spectra were recorded using the standard parameter sets as provided by Bruker with CDCl_3_ as the solvent. Chemical shifts are reported in δ units (ppm) and were referenced to the residual solvent residual peak (^1^H- 7.26 ppm for CDCl_3_; ^13^C: 77.0 for CDCl_3_).

Matrix-Assisted Laser Desorption/ Ionization Mass spectroscopy (MALDI-MS). A RapifleX MALDI TOF/TOF mass spectrometer (Bruker Daltonics, Bremen, Germany) equipped with a Smartbeam 3D laser was used in positive ion mode for all data acquisition. The DHB matrix is a 20 mg/mL solution in methanol. The La(III) complexes were solubilized in methanol and a freshly prepared DHB matrix. The solution was stirred with a Vortex-Genie 2 at a 1:2 (*v*/*v*) ratio, then spotted in triplicate onto 396-MTP Ground steel targets (Bruker Daltonics) using a dried-droplet technique in 1 µL aliquots. The ionization was performed at a laser power of 60% to 80%, and a total of 1000 laser shots were accumulated for each spectrum. The samples were studied in Reflector mode with a detector voltage of 2117 V, a digitizer frequency of 1.25 GHz, 1000 shots per pixel, a laser frequency of 5 kHz, and a mass-to-charge range of 100–2000 Da. The spectra were registered with FlexControl software (v4.0) and processed with FlexAnalysis software (v4.0) (Bruker Daltonics, Bremen, Germany). Mass calibration of the MALDI-TOF/TOF-MS was provided by a peptide mixture standard solution (Bruker Daltonics, Bremen, Germany).

Thermogravimetric analysis (TGA). The TGA measurements were conducted on a STA 449 F1 Jupiter (Netzsch, Selb, Germany). About 10 mg of each sample was weighed and heated in alumina crucibles, over the temperature range of 30 °C to 700 °C at a heating rate of 10 °C/min. Nitrogen gas was purged as an inert atmosphere at a flow rate of 50 mL/min.

Differential Scanning Calorimetry (DSC). The DSC measurements were conducted on a 200 F3 Maia DSC device (Netzsch, Selb, Germany). The apparatus was calibrated with indium. Around 9 mg of sample was heated in alumina crucibles with pierced and sealed shut lids at a heating rate of 10 °C min^−1^. Nitrogen was used as purge gas (flow rate 50 mL min^−1^). The melting enthalpy was calculated as the area under the endothermic peak in the first heating curve.

Infrared spectroscopy. The infrared spectral measurements were made in attenuated total reflectance mode (ATR) on a Bruker Vertex 70 FTIR spectrometer (Bruker Optics, Ettlingen, Germany) equipped with a Golden Gate device (diamond crystal, 45° incidence angle, single bounce, from Specac Ltd., Orpington, UK)). A total of 128 scans at 2 cm^−1^ resolution were averaged. The polarized ATR spectra were recorded by mounting a wire grid polarizer (Perkin Elmer, Shelton, CT, USA) between the IR source and the sample. OPUS 6.5 software was used to collect and to process the data.

UV-Vis and fluorescence emission spectroscopy. The UV-Vis absorption spectra were recorded with the Analytik Jena Specord Plus 5 spectrophotometer (Analytik Jena GmbH, Jena, Germany) using the integrating sphere. The fluorescence emission was measured using a Perkin-Elmer LS55 fluorimeter. The excitation-dependent emission spectra were recorded with an FS5 fluorimeter (Edinburgh Instruments, Livingston, UK). The emission was scanned from 350 to 750 nm, with the excitation light set between 270 and 400 nm, in 10 nm steps.

Electron spin resonance spectrometry (ESR). The ESR spectra were obtained at 25 °C using an EMX X-band (Bruker Nano GmbH, Berlin, Germany) spectrometer operating at 9.5 GHz, with 100 kHz field modulation. Other experimental parameters were: center field 3354 G, sweep width 200 G, receiver gain 40 dB, modulation amplitude 3 G, attenuation 15 dB. The spectra were recorded after 10 successive scans, without signal accumulation, to minimize noise. Rubbery film samples of different sizes were fixed in EPR Tissue Sample Cells (ATS Life Sciences Wilmad, Vineland, NJ, USA) according to the manufacturer’s instructions.

Wide-angle X-Ray diffraction (WAXD). The WAXD analysis was performed using a Bruker-AXS D8 Advance diffractometer (Bruker AXS, Karlsruhe, Germany), with a Bragg–Brentano goniometer. The XRD diffractograms were recorded using Ni-filtered Cu K_α_ radiation (λ = 0.1541 nm), over the 2θ range of 4–40°. The tube voltage was 40 kV and the tube current was 30 mA. The collected data were processed using the Bruker software Eva 11 and Topaz 3.1. The average distance between the folded chains of PDMS, *d*, was calculated according to Bragg’s law n⋅λ=2dsinθ. The average size of the ordered regions, *L*, was calculated according to the Scherrer equation FWHM=(0.9⋅λ)/(L⋅cosθ), where FWHM is the full width at half maximum and 0.9 is the value of the Scherrer factor. The degree of crystallinity was determined by dividing the integrated intensity of the crystalline reflection by the total area of the XRD signal.

Optical microscopy. The surface morphology of the samples, before and after photoaging, was analyzed using a Leica DM 2500 M optical microscope (Leica Microsystems, Wetzlar GmbH, Wetzlar, Germany) in polarized light, at room temperature, at magnifications of 200× and 500×.

Water contact angle measurements. The surface’s hydrophobicity was determined by measuring the static contact angles using the sessile drop technique at room temperature using CAM 101 Optical Contact Angle instruments (KSV Instruments Ltd., Helsinki, Finland). Images were recorded using a special optical system equipped with a CCD camera connected to a computer. The test liquid used for determinations was MilliQ water. A drop of liquid (~1 μL) was placed on the sample surfaces using a Hamilton syringe, and the image was immediately captured by the CCD camera and sent to the computer for analysis. All measurements were performed in triplicate, and the results were recorded as mean ± standard deviation.

Nanomechanical measurements. The force–displacement curves were recorded to extract the adhesion forces using an Ntegra AFM platform (NT-MDT Spectrum Instruments, Zelenograd, Moscow, Russia). The NSG10-type cantilever (TipsNano, Tallinn, Estonia) has a resonance frequency of 172 kHz and a spring constant of 3.2 N/m, which was calculated with John Sader’s method, based on the frequency response apex and the geometric characteristics (length of 125 μm and width of 27 μm).

## 3. Results and Discussion

### 3.1. Synthesis and Characterization of the Schiff Base Ligands, La(III) Complexes, and of Composites

The Schiff base ligands were prepared starting from two diamines possessing dimethylsiloxane/dimethylsilane units in their structures [32,33] and 2-hydroxy-3-methoxy-benzaldehyde by condensation reaction in acetonitrile, for 2 h at reflux (Scheme 1a). The products, named H_2_**L1** and H_2_**L2**, were isolated in high yields, 85.7–90.6%. Their structures were first confirmed by FTIR, ^1^H-NMR, and ^13^C-NMR spectroscopy (Figures S1–S6). The first evidence in the IR spectrum was the presence of the band at 1619 cm^−1^, characteristic of the imine group. In the ^1^HNMR spectra of the ligands, the chemical displacements for the imine protons appeared at 8.60–8.66 ppm, while those for the carbon atom involved in the imine bond formation appeared at 164.04–164.11 ppm in ^13^C-NMR spectra. The La(III) complexes were prepared by mixing the ligand and La(NO_3_)_3_ solutions in methanol at 50 °C overnight. During the reaction, a precipitate was formed, which was isolated by filtration, washed with cold methanol, and dried. The formation of the complexes was confirmed by IR, ^1^H-NMR, and MALDI-MS spectra (Figures S7–S12).

In the FTIR spectra of the La(III) complexes (Figures S7 and S8), the blueshift of the imine unit from 1619 cm^−1^ in the ligand to 1634/1637 cm^−1^ confirms the coordination of the phenolic oxygen/nitrogen atom to La(III) ions. The coordinate nitrate groups are recognized by the vibrations at 1506 (ν_4_), 1304 (ν_1_), 1114 (ν_2_), and 857 cm^−1^ (ν_6_) [37]. The broad OH stretching vibration centered at 3420 cm^−1^ in ligands and complexes is assigned to water molecules existing in the coordination sphere of La(III) ions. In the ^1^H-NMR spectra, the chemical shifts of the imine protons appear at 8.93–8.95 ppm, confirming the involvement in the coordination of La^3+^ ions. The MALDI-MS spectra show the molecular ion [M]^+^ at *m*/*z* 961.261 for LaSiOSi and [M + Na]^+^ at *m*/*z* 928.15, which is specific to LaSi.

The hybrid materials prepared by incorporating 2.5% La(III) complexes into a PDMS matrix, using TEOS as a crosslinker and DBTL as a catalyst, were isolated as free-standing films. The maturation occurred under normal conditions (RT, laboratory air conditions). The films thus prepared are stable under normal conditions. They exhibit high flowability and surface uniformity, without phase separation (see Figures S13–S16 for photographs and SEM images of the surfaces and cryo-fractured films).

ATR-FTIR spectroscopy of PDMS-based nanocomposites. In hybrid LaSi_PDMS and LaSIOSi_PDMS films, the absorption band of coordinated water has disappeared, proving they are anhydrous. The characteristic ν_as_(Si-O-Si) vibration of crystalline domains in pure PDMS [38] is narrow and is observed at 1082 cm^−1^, but becomes a little broader in composite films, with a blueshift at 1084 cm^−1^ in LaSi_PDMS and a redshift at 1073 cm^−1^ in LaSiOSi_PDMS. A quick comparison of the infrared spectra of La(III) complexes shows that almost all bands changed markedly after blending with PDMS. The initially intense ν(C=N) and ν(C=C) vibrations at 1634/1618 and 1602 cm^−1^ are completely missing for both composites, and the ν(C=C) bands at 1573 and 1496 cm^−1^ of LaSi became very strong. These issues lead to the hypothesis that the La(III) complex must be oriented relative to the pure PDMS chain, and some aromatic rings are confined to particular positions.

**Figure 1:**
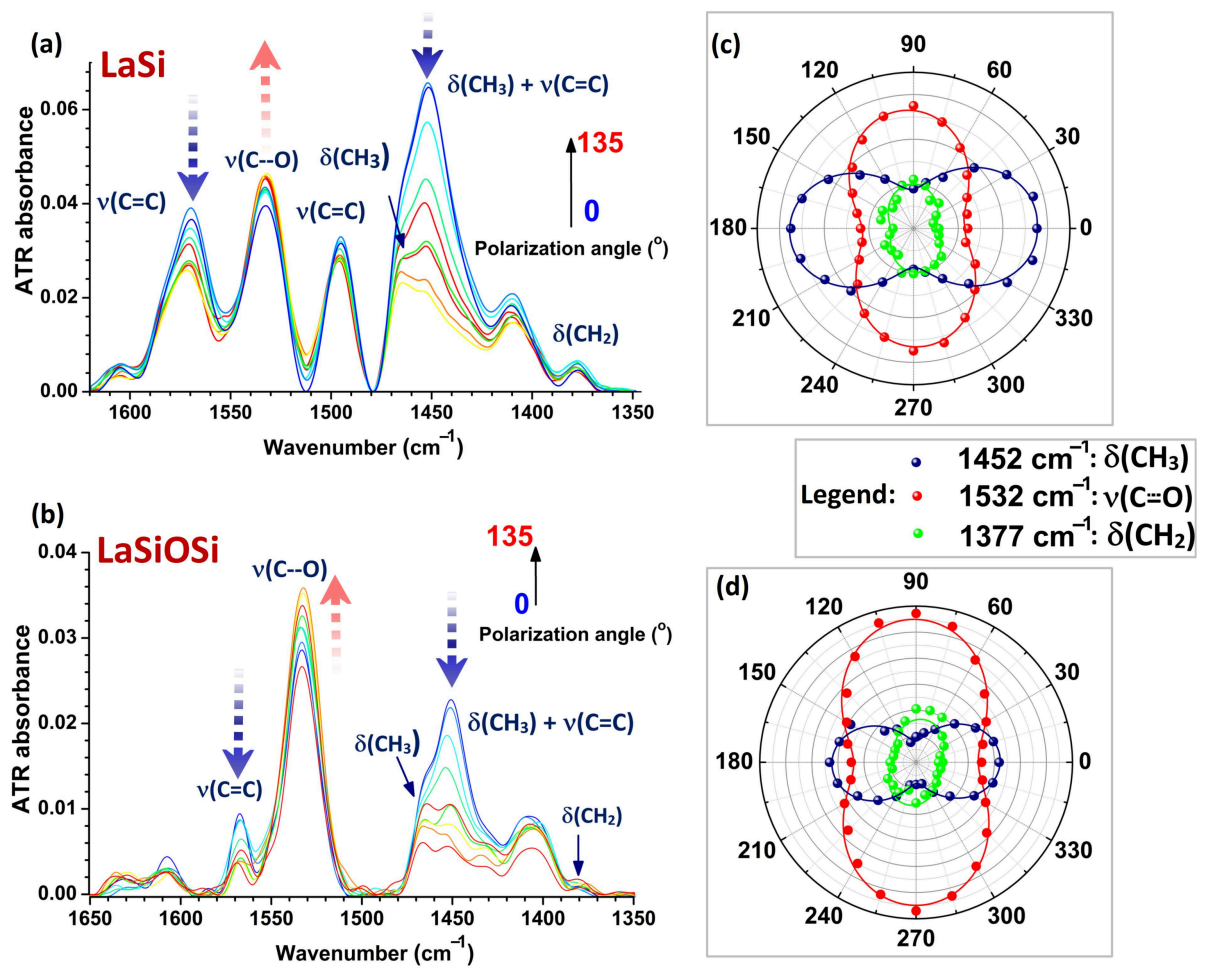
Polarization–dependent ATR-FTIR spectroscopy for polarization angle varying between 0° and 135°: normalized spectra of LaSi (**a**) and LaSiOSi ligands (**b**) in the composite films; (**c**) polar plots of three key vibrations of LaSi_PDMS; (**d**) polar plots of the same vibrations for LaSiOSi_PDMS. The blue and red arrows in (**a**) and (**b**) indicate the vibrations whose transition dipole moments are oriented parallel and perpendicular, respectively, on the axis of the spacer of the ligand.

The molecular orientation of the La(III) complexes was determined using polarized ATR-FTIR spectroscopy. Figure 1 shows the 1660–1350 cm^−1^ region of the ATR-FTIR spectra of LaSiSi_PDMS and LaSI_PDMS, when the polarization angle of the IR beam varied from 0° to 135°. The infrared spectra of the fillers were obtained by subtracting the PDMS spectrum from the blend at each polarization angle. The arrows show the variation in band intensity as a function of the polarization angle, indicating the degree of molecular anisotropy of the La(III) complexes within the PDMS matrix. The right part of Figure 1 shows the polar representations of the band intensity of three important vibrations of the fillers: bending of CH_2_ and CH_3_ groups, at 1377 and 1452 cm^−1^, respectively, and the phenolate stretching vibration at 1532 cm^−1^ [39]. The band at 1452 cm^−1^, which shows strong parallel dichroism, is in fact a combination of the CH_3_ bending and of aromatic C=C stretching vibrations [39].

By judging from the parallel dichroism of the aromatic ν(C=C) band at 1568 cm^−1^, we observe that the aromatic rings have a low degree of freedom, especially those linked to the ester group. So, the aromatic ester adopted a certain position with reference to the -Si(CH_3_)_2_-O-Si(CH_3_)_2_- segment of the PDMS chains, a position that is imposed by the planarity of the phenyl rings. This fact suggests that the transition dipole of the 1452 cm^−1^ vibration is oriented along the main axis of the flexible spacer and of the PDMS chain. The perpendicular dichroism of the spacer’s methylene groups is very weak and irregular, confirming the perpendicular orientation of the transition dipole to the main chain axis.

The anisotropies of absorbances discussed above show a double-lobed elliptical symmetry in Figure 1c,d, with a variation period of 180°. The anisotropy of aromatic vibrations is extremely regular, which confirms that the Ph–C(=O)-O-CH_2_ fragment of the spacer is aligned parallel with the ATR crystal–film interface. This further suggests that the filler is not randomly dispersed within the matrix but is constrained by the flexible spacer to orient along the PDMS main chain and near the siloxane bonds.

### 3.2. Accelerated Weathering of PDMS Composite Films

#### 3.2.1. Thermogravimetric Analysis

Table 1 lists the characteristics extracted from the TG analysis curves, such as: the temperatures corresponding to 5 wt. % (*T*_5%_), 30 wt.% (*T*_30%_) and 50 wt. % (*T*_50%_) mass losses; the temperature corresponding to the maximum rate of decomposition (*T_peak_*); percentage of mass loss corresponding to each thermal degradation stage, and the mass of residue at the end of the thermal degradation process (W). The thermograms and their corresponding first derivatives (DTG) for the initial samples and for those exposed to 600 h of photoaging are presented in Figure S18. The static heat resistance index (*T_s_*) was calculated with Equation (1) and used in estimating the thermal stability of the structures [40]:

*T_s_* = 0.49 [*T*_5%_ + 0.6 (*T*_30%_ − *T*_5%_)] (1)

As shown in Figure S18 and Table 1, the initial structure thermally decomposes in four (PDMS and LaSi_PDMS) and in three stages (LaSiOSi_PDMS), with most overlapping due to the complexity of the decomposition process. The neat PDMS is the most thermally stable of all the samples, with *T_s_* = 195 °C, followed by LaSi_PDMS (*T_s_* = 194 °C) and LaSiOSi_PDMS (*T_s_* = 178 °C). The onset of weight loss decreases by almost 40 °C in composite films. The *R* values are comparable (20.09%, 23.27% and 21.11%, respectively). Thus, the introduction of La(III) complexes led to a gradual decrease in the thermal stability of the initial samples, with the lowest value observed for LaSiOSi_PDMS, which contains the flexible Si-O-Si link. Table 1 also shows that the mass loss values (W) may vary across thermal degradation stages, due to the introduction of La(III) complexes. Firstly, the structures have degraded via random intra- or intermolecular redistribution reactions among siloxane bonds within the polymer matrix, proceeding through the formation of four-center transition states that further yield cyclic volatile siloxane rings [41,42]. The random scission mechanism causes a significant decrease in molecular weight from the onset of degradation, resulting in a broadened molecular weight distribution. In addition, the low-molecular weight linear compounds that are linked to the bulky end-caps decompose in the higher temperature stages of thermal degradation, due to their inability to take part in the ring-closure reactions. Since the samples are sol-gel crosslinked structures, they yield low concentrations of cyclic siloxane oligomers and higher concentrations of linear siloxane entities, such as dimers or trimers, which decompose together with the bulky end-caps, resulting in a complex overlapping thermal decomposition process. Compared to the thermal decomposition of initial samples, the samples photoaged for 600 h exhibited considerable thermal stability (*T_s_* = 234 °C for neat PDMS, 254 °C for LaSi_PDMS and 217 °C for LaSiOSi_PDMS). Also, PDMS and LaSi_PDMS thermally decompose in two stages, whereas LaSiOSi_PDMS decomposes in three stages, with DTG peaks merging into a single peak and the structures tending to behave as monocomponent systems. Furthermore, the *R* values are also significantly higher for the photoaged structures, except for the initial neat PDMS (17.88% for PDMS, 37.6% for LaSi_PDMS and 26.54% for LaSiOSi_PDMS). All these aspects indicate that photoaging leads to extensive crosslinking.

#### 3.2.2. DSC Study

**Figure 2:**
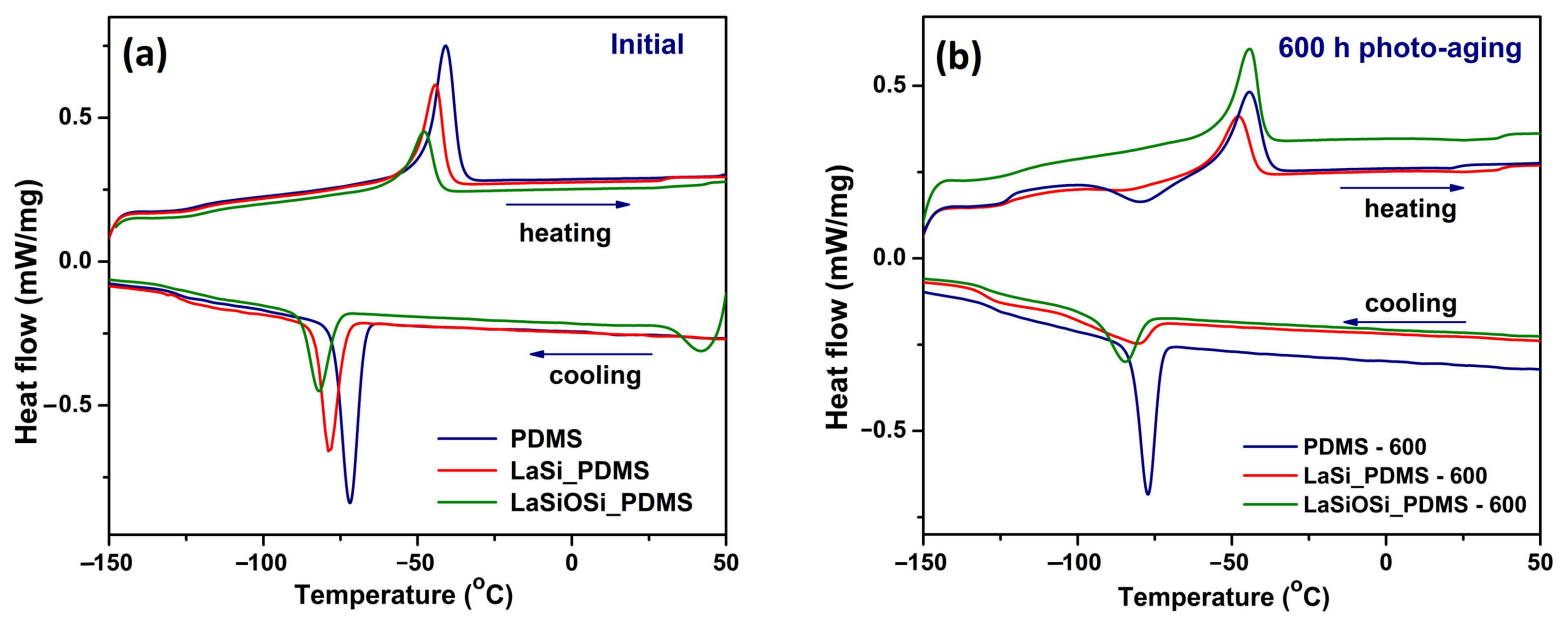
DSC thermograms of the melting and crystallization cycles for PDMS, LaSi_PDMS, and LaSiOSi_PDMS before (**a**) and after 600 h of photoaging (**b**).

Figure 2 presents the DSC thermograms of the initial and photoaged samples at 600 h, during the cooling and second heating cycles, recorded at 10 °C/min. The DSC curves for the entire series of photoaged PDMS-based films are illustrated in Figure S19. In Figure 2, the typical PDMS transitions are observed: the glass transition around T_g_ = 120 °C, melting at T_m_ ≅ −45 °C, and crystallization around T_cr_ ≅ −80 °C. The crystallinity degree (χ_c_) was calculated with the equation (Δ*H*_m2_/Δ*H*_literature_) × 100, where Δ*H*_m2_ is the enthalpy of the melting profile corresponding to the second heating run, and Δ*H*_literature_ = 61.3 J g^−1^ [43].

The addition of La(III) complexes to PDMS lowers the melting temperature from −41 °C for PDMS down to −48 °C for LaSiOSi_PDMS (Table S1), which may be due to the formation of smaller and imperfect crystallites [44]. This is because the silane/siloxane portion of PDMS slows chain motion and retards crystallization. Also, the melting enthalpy decreases from neat PDMS (21.66 J/g) to LaSiOSi_PDMS (11.48 J/g), indicating a lower crystalline fraction. Because PDMS alone has a 35% degree of crystallinity, the percentage is practically halved in LasiOSi_PDMS (18%) (Table S1), which can be explained by the strong anchoring of LaSiOSi to PDMS chains via intermolecular interactions, thereby reducing the chains’ mobility and their ability to orient. Thus, the cold crystallization temperature is shifted downward by 10 °C in LaSiOSi_PDMS (−82 °C) compared with neat PDMS (−72 °C).

The melting temperature values did not change much for LaSi_PDMS (−44 °C to −43 °C) and LaSiOSi_PDMS (−48 °C) under photoaging. The *T_m_* value of PDMS lowered from −41 °C to −45 °C (Figure S19), because the PDMS crystallites have become thinner [45] as a result of extended crosslinking. The melting enthalpy decreased drastically in the first 200 h for PDMS alone (from 21.66 J/g to 12.2 J/g) and LaSi_PDMS (from 15.58 J/g to 11.23 J/g, Table S1), reflecting reduced crystallinity and the development of a crosslinked architecture. The crystallization temperature is mainly affected by photoaging in PDMS, where it decreased from −72 °C to −77 °C, because shorter chains are more easily arranged into crystals. Crosslinking in neat PDMS increased the *T_g_* value after 600 h of photoaging from −123 °C to −121 °C, whereas *T_g_* remained the same in the composites. The crystallization enthalpy, already higher in composites than in neat PDMS, increased further due to photoaging, with a great jump after the first 100 h for PDMS alone. As expected, the crystalline fraction is lower in all photoaged samples, with the largest decrease occurring in the first 200 h for PDMS (of 37%), from 35% to 20% at 200 h (Table S1).

#### 3.2.3. Surface Chemistry Revealed by Infrared Spectroscopy

**Figure 3:**
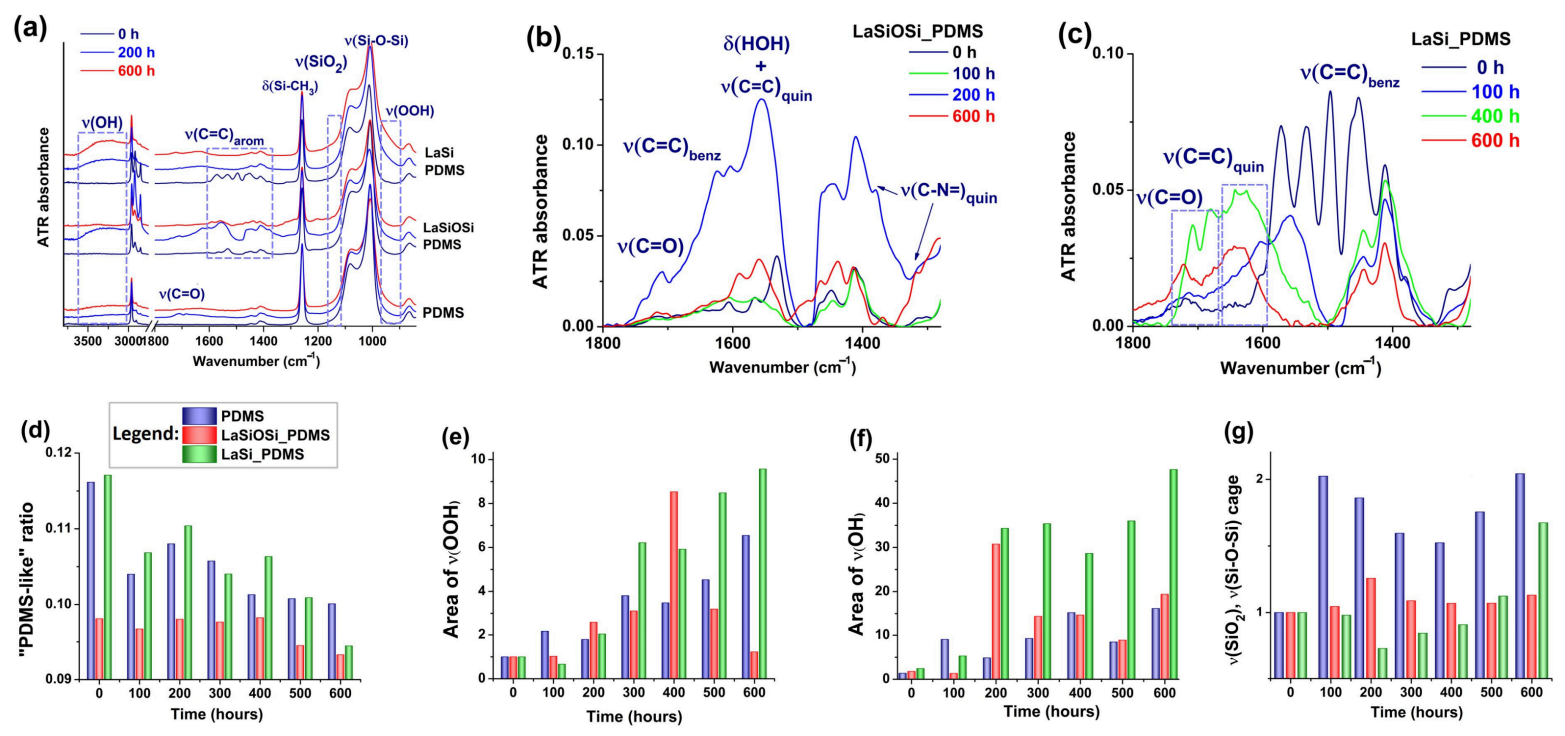
ATR-FTIR spectroscopy of photoaged PDMS-based composites: (**a**) ATR-FTIR spectra of initial, intermediary, and final irradiation times; (**b**) spectral variation of the LaSiOSi ligand in composite film; (**c**) spectral variation of the LaSi ligand in composite film; (**d**–**g**) relative variations in ATR integrated absorbance values for: (**d**) Si(CH_3_)_2_/(Si(CH_3_)_2_ + SiOSi)) ratio; (**e**) ν(OH/OOH) (3800–3000 cm^−1^); (**f**) ν(OOH) of peroxides + silanols (940 cm^−1^); (**g**) SiO_2_/cage ν(Si-O-Si) (1190–1130 cm^−1^).

The surface chemistry of PDMS composites was investigated using infrared spectroscopy to determine the structural changes induced by photoaging. Figure 3a presents the ATR-FTIR spectra of the neat PDMS, LaSiOSi_PDMS, and LaSi_PDMS films before photoaging, after 200 h, and after 600 h of photoaging, respectively. The complete spectral evolution of the three samples is presented in Figure S20. In a global analysis, artificial sun exposure produced a general decrease in intensity, and a shift and broadening of the absorption bands assigned to ν(Si-C) (785 cm^−1^) and of ν(Si-O-Si) at 1009 and 1075 cm^−1^ [35]. The development of the large ν(OH/OOH) band in the 3700–3100 cm^−1^ region was observed in all samples, but its profile and intensity varied with filler and photoaging time.

The broad band between 1760 and 1600 cm^−1^ covers overlapped signals from: ν(C=O) of acids, peracids, and formate esters (1708 cm^−1^), ketones (1683 cm^−1^), δ(OH) from water (1640 cm^−1^), and ν(C=O) of silyl ketones (1630 cm^−1^) [19]. These signals result from reactions between PDMS and the atmospheric oxygen. The spectral variation of LaSiOSi and LaSi ligand within composite films, given as a close-up in Figure 3b,c, shows the disappearance of the benzenoid ν(C=C) vibrations around 1606, 1567, and 1532 cm^−1^ after 100 h of photoaging and appearance of semiquinoid ν(C=C), ν(C-N=) and ν(C-N) vibrations at 1556, 1484, 1368, and 1204 cm^−1^, respectively [46,47]. Weak bands at 1625, 1380, and 1314 cm^−1^ are clear in the spectra of LaSiOSi_PDMS, and they are assigned to ν(C=C) and ν(C-N=) vibrations in full quinoid rings [46,47]. Further irradiation led to the complete depletion of quinoid signals.

The profile of the ν(Si-O-Si) band (1009/1075 cm^−1^) remained relatively the same in all samples. Still, differences between initial and photoaged samples are clear: redshifting and an increase in intensity of the two peaks, an enhancement of the valley around 1060 cm^−1^, and two broad absorptions that flank the base of the ν(Si-O-Si) band. The 1060 cm^−1^ peak is in the region assigned to ν(SiO_2_) in “network”-like crosslinked structures, where the Si-O-Si angle is of ≈144° [48]. The development of networked ν(SiO_2_) is lower in composite films than in neat PDMS. The first flanking band is in the 1190–1130 cm^−1^ region, corresponding to ν(SiO_2_) with a larger Si-O-Si angle (≅150°) and forms “cage”-like crosslinks [48]. The second flanking band is the long tail in the 970–900 cm^−1^ domain, specific to ν(OO) of hydroperoxide groups [19,39] and to silanol/siloxanol Si-OH units. The existence of “cage” and “network”-like crosslinked structures indicates that a fraction of hydroxyl groups is bonded directly to the Si atoms. New bands at 2925, 2857, 1462, and 1214 cm^−1^ appear clearly in all photoaged specimens and are given by Si-O-CH_2_-, Si-CH_2_- species, and Si-CH_2_-CH_2_-Si linkages [39,49].

The demethylation and degradation of the Si(CH_3_)_2_ − O-Si(CH_3_)_2_- fragment can be analyzed by employing the “PDMS-like ratio” Si(CH_3_)_2_/(Si(CH_3_)_2_ + SiOSi)), where Si(CH_3_)_2_ is the area of the δ(Si-CH_3_) band (1259 cm^−1^), and SiOSi is the area of the of ν(Si-O-Si) doublet [50]. Figure 3d shows that decomposition of methyl groups took place in every sample, but the most regular trend is observed in native PDMS, and the highest rate is in LaSi-PDMS. Development of peroxides, peracids, and adsorbed water in their immediate environment (Figure 3e,f) is the highest in LaSi_PDMS, for which the peroxides are continuously generated. The threshold at about 300 h of photo-aging marks the maximum oxidation of the pendant methyl groups, at which acids and peroxyacids were formed. These processes were accelerated in LaSi_PDMS, as the threshold falls at about 200 h. The disappearance of acidic groups after the threshold suggests they further participate in secondary reactions, giving rise to ketones, -O–O- and Si-O-Si bridges, or they can be removed from the surface as low molecular compounds.

After 400 h of photoaging, the expansion of the caged silicon oxide network is exponentially amplified in PDMS and LaSi_PDMS (Figure 3g), which correlates with the accelerated decomposition of methyl groups (Figure 3b). It is important to emphasize the inverse relationship between the intensities of ν(OH) and ν(C=O), because the magnitude of ν(OH) is influenced by ambient relative humidity (RH), which strongly affects photoreactions and the generation of oxygen-containing groups [28].

#### 3.2.4. Optical Properties of Photoaged PDMS-Based Nanocomposites

**Figure 4:**
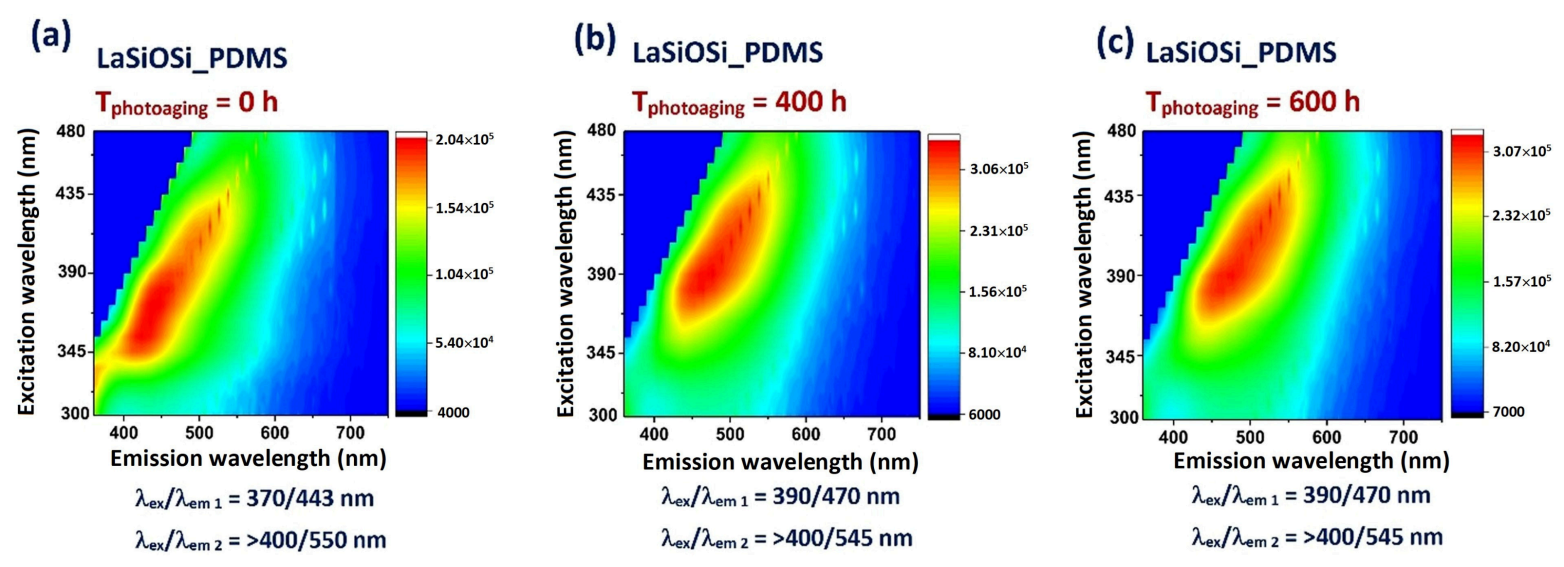

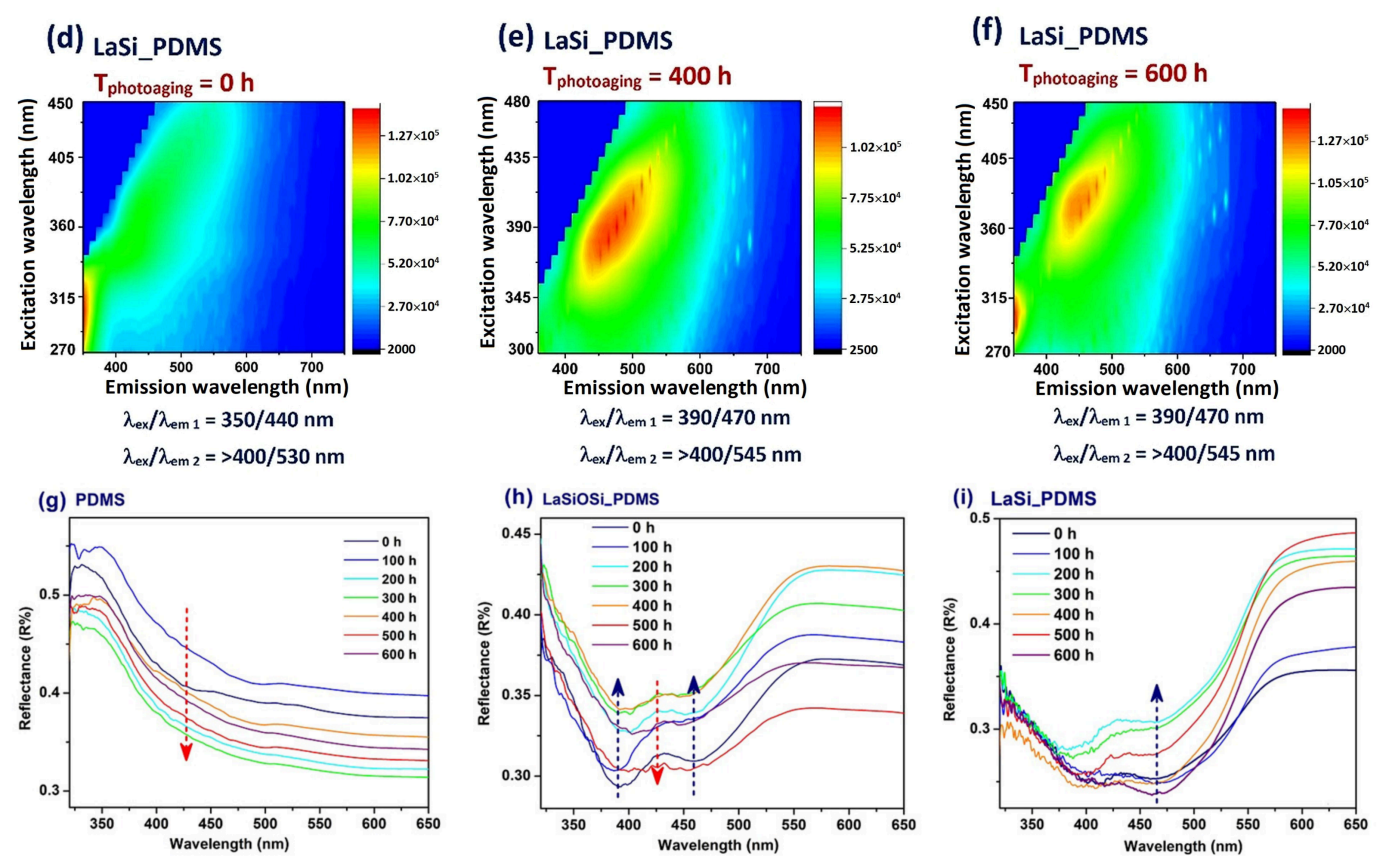
Effects of accelerated UV-aging on the optical properties of LaSi/LaSiOSi_PDMS films: (**a**–**c**) EEM spectra of LaSiOSi_PDMS at three photoaging times; (**d**–**f**) EEM spectra of LaSi_PDMS at three photoaging times; (**g**–**i**) Reflectance spectra for neat PDMS (**g**), LaSiOSi_PDMS (**h**), and LaSi_PDMS (**i**). The red arrow in (**g**–**i**) indicates the increase in the reflectance. The blue arrows indicate a decrease in the reflectance.

We conducted optical investigations of photoaged nanocomposites to confirm the presence of oxidation products on their surface. Both La(III) complexes are fluorescent, and the original ligand-centered emission maximum in the solid state is found in the red zone at 628 nm (Figure S20). Immersion in PDMS quenched the red emission and left a residual fluorescence composed of a blue band around 460 nm and a green band around 540 nm. Photoproducts with a conjugated structure will, in theory, redshift the fluorescence emission and the absorption. To test this assumption and map the changes during photodegradation, we analyzed the emission of composite films over a wide range of excitation wavelengths. The EEM spectra at the initial, intermediate, and final photoaging times are shown in Figure 4a–f. All the spectra look similar because they share a wide fluorescence peak. The emission shifts from blue to green, combining the initial two peaks, at λ_ex_/λ_em_ = 370/440 nm and at λ_ex_/λ_em_ ≥ 400/530–550 nm. The identified species are: ketonic and/or anionic structures, for λ_em_ = 500–530 nm, quinoids for λ_em_ > 530 nm, and probably enol tautomers, for λ_em_ ≈ 440 nm [34,51,52]. The fluorescence spectra of photoaged LaSi_PDMS show the increase of the green band around 558 nm and the appearance of a new one around 485 nm (Figure S20b). There is no photobleaching for any of the ligands, and the photoproducts appear to be stable over time, or a regular interconversion between several forms takes place.

The reflectance spectra of LaSiOSi and LaSi contained within PDMS, presented in Figure 4h,i, initially consist of two bands, around 330 nm (n ⟶ π* transition) and 460 nm, which are typical for complexes of Schiff base ligands [34,51,52]. After photoaging, all films showed a broad band centered at 430 nm, along with a reduction in the ligands’ absorption. The reflectance of transparent PDMS decreased after photoaging. New bands at 312/315 nm and 260 nm are associated with the formation of ketones and aldehydes, as well as with the silica network on the surface [53].

#### 3.2.5. Structural Characterization by WAXD

**Figure 5:**
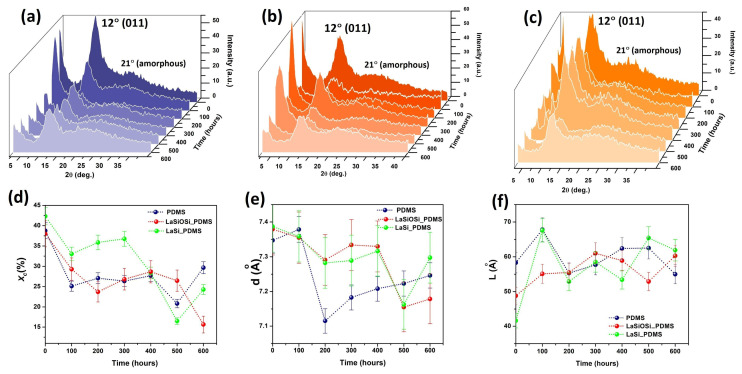
Crystallinity of PDMS-based films upon photoaging: WAXD profiles of native PDMS (**a**); LaSiOSi_PDMS (**b**); LaSi_PDMS (**c**); (**d**) Variation of crystallinity degree (*X_c_*); (**e**) Variation of the interlayer *d*-spacing of the PDMS matrix; (**f**) Variation of crystallite size (*L*).

The structural organization of PDMS chains upon photo-aging, in the absence and in the presence of LaSi and LaSiOSi, was analyzed by WAXD. The consecutive X-Ray diffractograms of crosslinked PDMS and composite films are shown in Figure 5a,c. The 2D ordered domains of native PDMS are in the form of lamellae with folded chains [51] and are confirmed in the X-ray spectra of all three specimens by the peak at 2θ ≈ 12° (011). The composite films show this peak at roughly the same position, but with lower intensity, suggesting that the La(III) complexes did not alter the normal folding of the PDMS chains in a lamella. The amorphous peak at 2θ ≈ 21° is correlated with the distance between silicon atoms in the PDMS chains [54] and has the same broad profile in the initial crosslinked PDMS and composite films.

The WAXD pattern of initial LaSiOSi_PDMS (Figure 5b) contains a peak at 6.92° 2θ (1.27 nm). LaSi_PDMS (Figure 5c) shows two diffraction peaks in the low-angle region. As a result of photo-aging, the intensity of the main PDMS peak at 2θ = 12°2θ became less defined and decreased in all three specimens, suggesting a slight decrease in crystallinity (Figure 5d). Its small shift to higher angles is due to the expansion of the crystalline lattice. The plot of degree of crystallinity vs. photoaging time (Figure 5d), which closely follows the DSC data, indicates that the crystalline regions are significantly affected at longer irradiation times. We suppose that the LaSi filler, which is more hydrophobic than the matrix, has preferred to partially self-assemble, so that it became intercalated between the PDMS chains, in the amorphous regions, and within the PDMS lamella. LaSiOSi appears to be well dispersed within the PDMS matrix, owing to its structural compatibility with poly(dimethylsiloxane). The preferential alignment of the fillers with the PDMS chains has been previously observed by polarized infrared measurements.

The interlamellar distance *d* of the composite films decreased due to photo-aging (Figure 5e), whereas that of the unfilled PDMS varied irregularly. The size of PDMS lamellae in composite films (Figure 5f) increased under UV irradiation, due to the immobilization of siloxane chains from the edges of the existing lamellar domains by interaction with the LaSi and LaSiOSi fillers. As the FTIR data showed that crosslinking began after ≈300 h for LaSiOSi_PDMS, the fluctuations in *L* values after 300 h correlate with those observed in the degree of crystallinity in the DSC data. One concludes that after 300 h of photo-aging, the crystalline structure of the PDMS matrix becomes “frozen” due to the expansion of the silica network and crosslinking by ethylene bridges.

#### 3.2.6. Electron Spin Resonance Measurements

**Figure 6:**
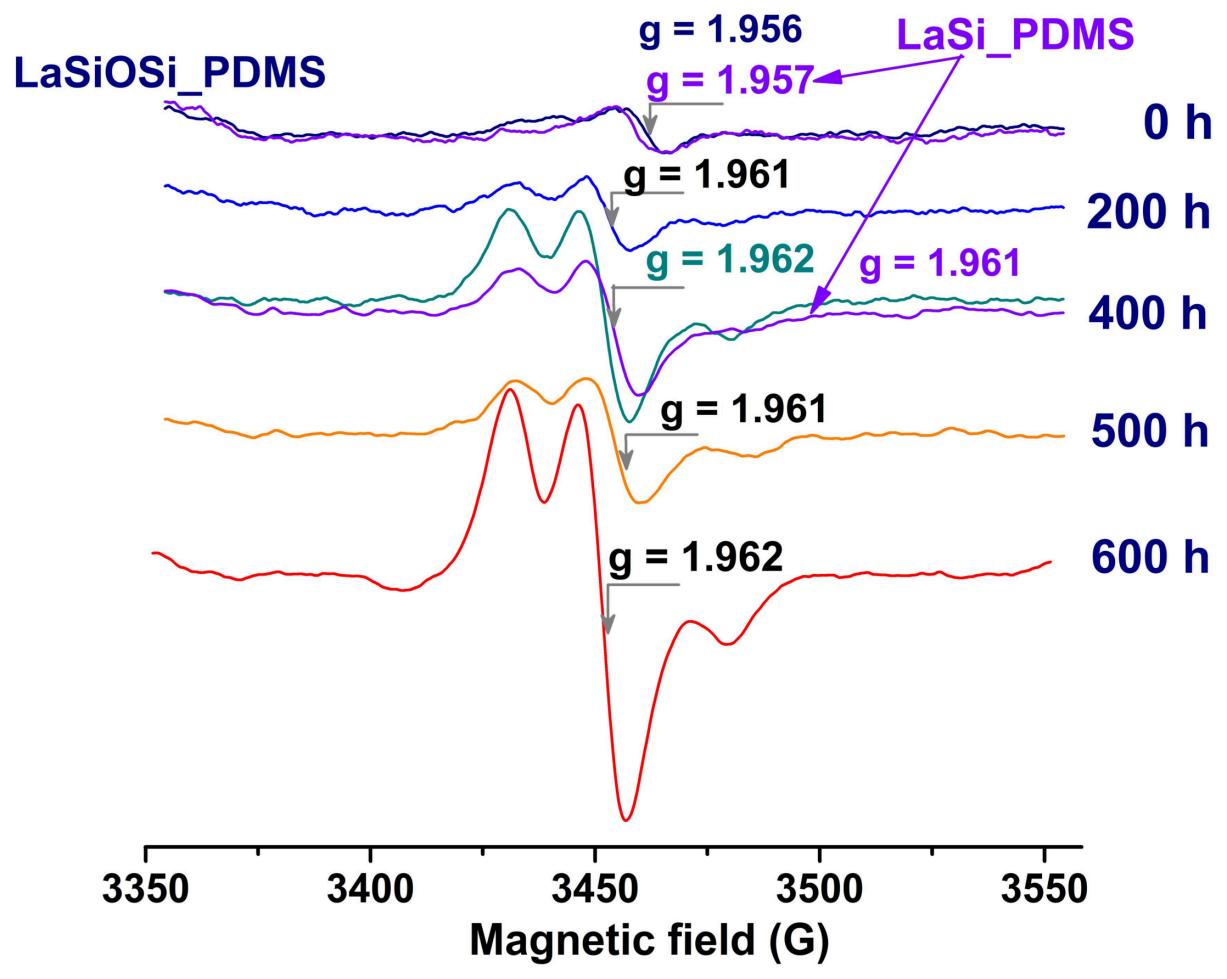
Representative ESR spectra of the photoaged PDMS-based composites and the corresponding g values.

Representative ESR spectra of initial and photoaged PDMS-based composites are shown in Figure 6. The initial LaSiOSi_PDMS and LaSi_PDMS films exhibit a very weak, nearly symmetric signal, with comparable intensities, due to the manufacturing process. The *g* values are 1.962 and 1.957, respectively. The peak-to-peak line width is 12.2 G for both. Changes in ESR signals during photoaging indicate the formation of metastable organic free radicals, most probably from PDMS chains, and secondary paramagnetic species. At least two radicals were stable over time. The ESR spectra of LaSiOSi_PDMS and LaSi_PDMS exhibit similar profiles and intensities, suggesting that the radicals are fundamentally similar. At irradiation times below 200 h, the asymmetry of the ESR signal is given by the splitting of the up-peak into two maxima at 3431.83 and 3448.16 G. The down-peak is at 3457.9 G and splits at higher photoaging times, the second peak being at 3479.94 G. The signals’ intensities increase during photoaging due to the increase in the unpaired spin density related to the higher oxidation level, with some fluctuations in between. The lower ESR signals at 500 h of irradiation are due either to the conversion of cation radicals in PDMS or fillers into spinless species, or to the elimination of volatiles. The *g*-factor values show a clear upward trend, reaching 1.962 at the end of the experiment. FTIR analysis revealed peroxide bands, supporting the conclusion that the observed ESR signals arise predominantly from peroxide radical centers. Similar ESR features have been reported for gamma-irradiated perfluorinated polymers in oxygen-containing environments, confirming the association with peroxide radicals [55].

### 3.3. Physical Characteristics of the Top Surface Layer: Morphology, Wettability and Adhesion Forces

#### 3.3.1. Surface Morphology

**Figure 7:**
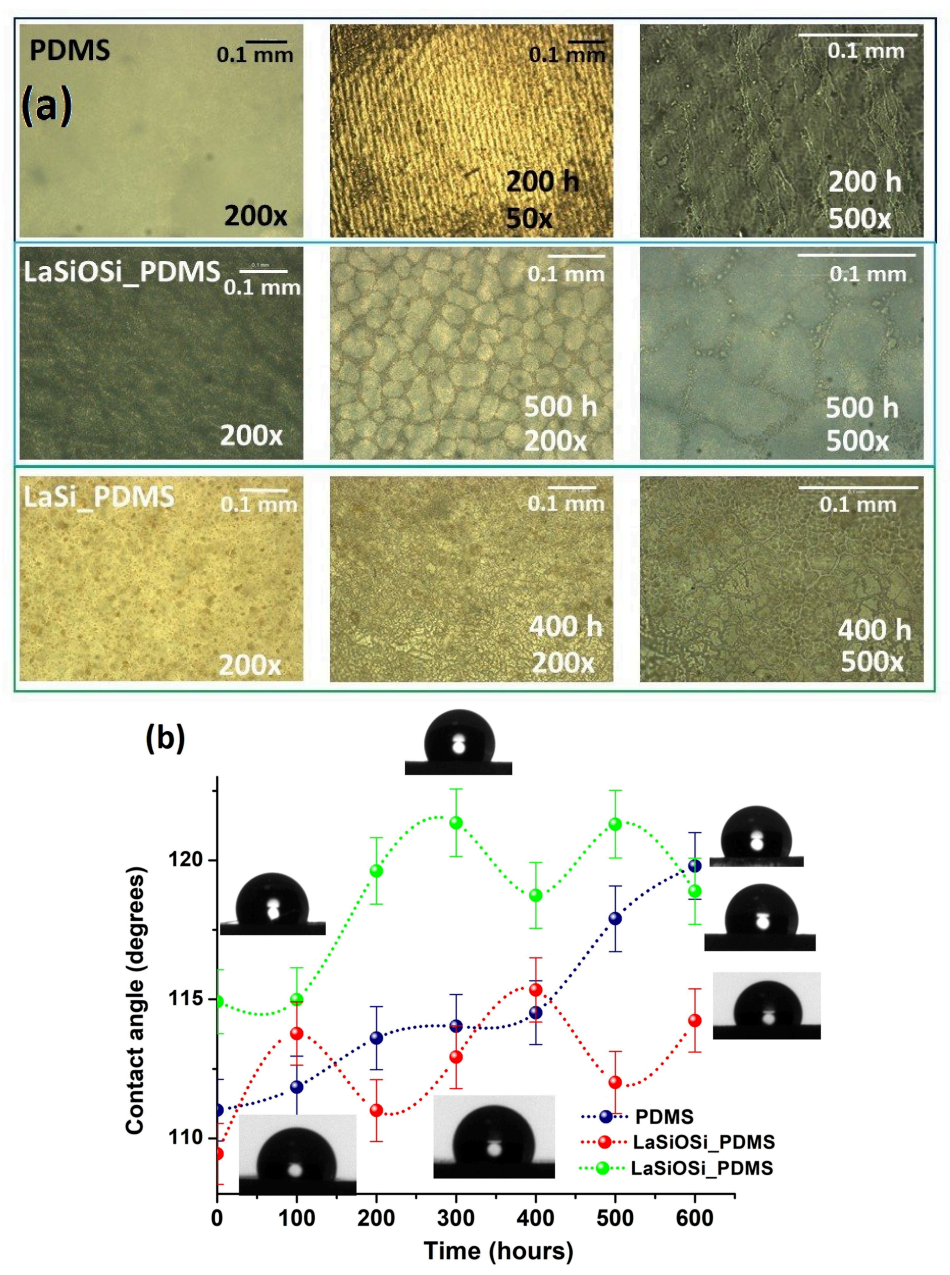
Effect of photo-aging time on the surface characteristics of PDMS films: (**a**) POM micrographs of initial and intermediary photoaged films; (**b**) contact angle of water and several optical images of water droplets.

The morphological changes of the PDMS composites induced by photoaging were first analyzed by polarized optical microscopy (POM). The neat PDMS exhibits a smooth and structureless surface (Figure 7a), which becomes patterned in stripe domains after 200 h of photoaging of ~25 μm width and with a periodicity of ~60 μm. Their micrometer-scale order suggests that the PDMS has lost mass during photoaging and air-drying. This mesostructuration is driven by phase segregation between hydrophilic silica domains and hydrophobic chain segments at specific RH values [56,57]. Formation and condensation of silanol groups, influenced by the water molecules in their vicinity, are essential to drive the exclusion of the hydrophobic segments with, e.g., -CH_2_-CH_2_- bridges, from the hydrophilic silica network. This means the medium is quite fluid and the ambient temperature is appropriate, allowing the non-volatile chain fragments and hydrophobic segments to align and self-assemble into supramolecular organizations.

No such alignment was observed for either LaSiOSi_PDMS or LaSi_PDMS. At low magnification, the surface of the initial LaSiOSi exhibits a wrinkled pattern, but the string-like crystalline structures are aligned in a certain direction. Regardless of irradiation time, the morphology changed to a homogeneous sea-island type, and the islands’ regular shape gradually became irregular as photoaging time increased (Figure 7a, middle). Smaller islands with multiple cracks are seen in the inter-island space, and their number has grown over time. The smooth surface of the initial LaSi_PDMS shows a macroscopic phase separation after photoaging. The dimpled shapes of varying sizes, without a specific directional pattern, are set on a glassy and cracked base, indicating some degree of plastic flow in the material before drying. The distribution of dimple size indicates multiple coalescence events, in which the silica network regularly fractures, releasing various photoproducts, oligomers, and debris to the surface through these fractures.

#### 3.3.2. Static Water Contact Angle

The measurements were performed using MilliPore water at room temperature, with the average of three independent contact angles measured on different parts of the films. Figure 7b shows that the static water contact angles (WCAs) exceed 100° for all photoaging times, indicating that the PDMS-based composites are hydrophobic. The neat PDMS has an initial WCA of 111° that agrees with the literature [58]. The surface becomes more hydrophobic as UV exposure time increases, reaching 119.8°, a value that correlates with surface roughness and the growing stripe pattern. The same results are observed for LaSi_PDMS: WCA increases from an initial value of 114.92° to around 120° when the photoaging time exceeds 300 h. These oscillations indicate the absence of major structural changes; hence, the silica layer is stable and permanent, and there is an equilibrium between the crack formation and the migration of unoxidized PDMS from the bulk. The WCA values of LaSiOSi_PDMS are 114°, corresponding to a smooth and granular structure.

#### 3.3.3. Local Nanomechanical Characteristics

**Figure 8:**
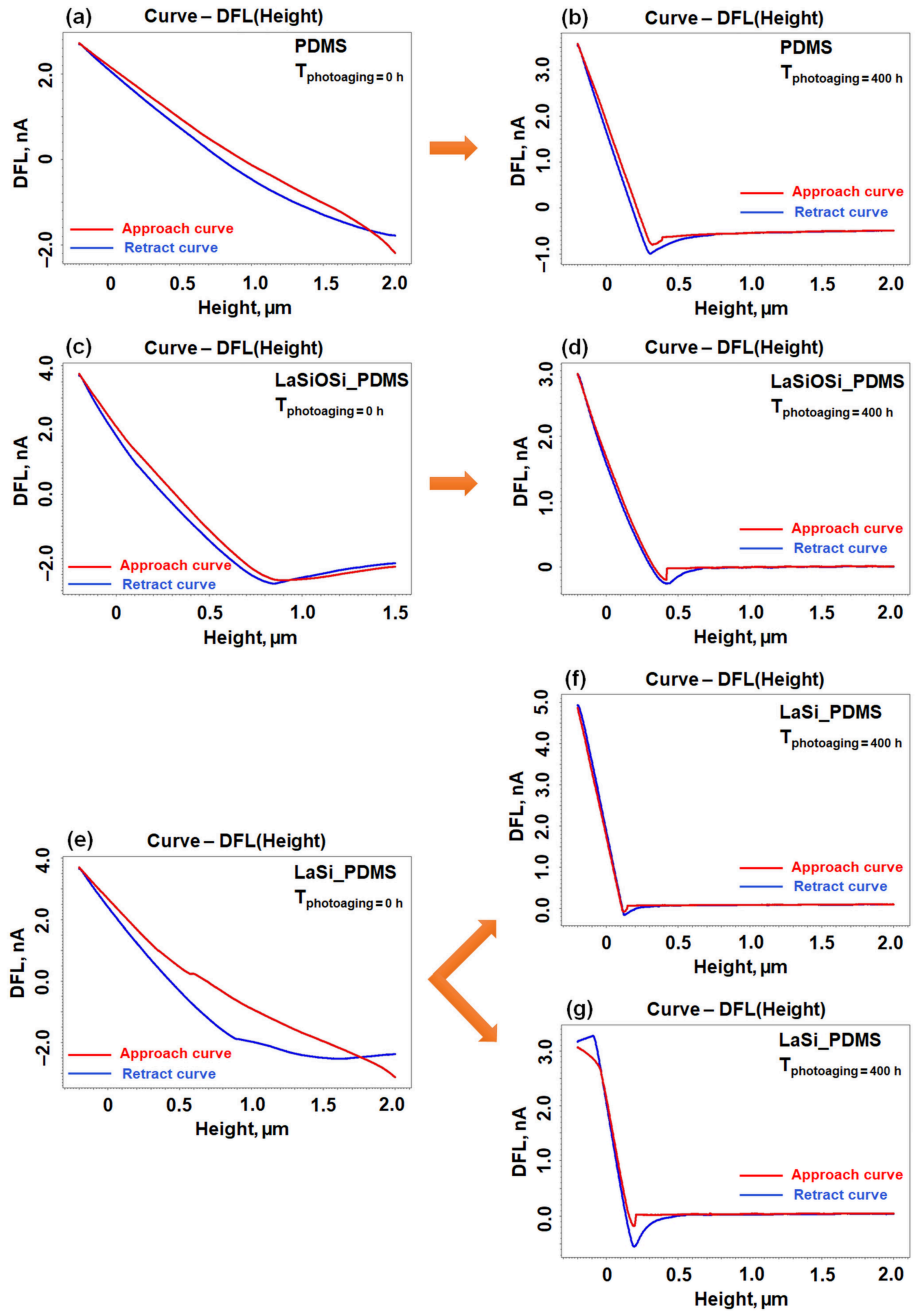
Representative DFL-height curves recorded for PDMS before photoaging (**a**) PDMS after 400 h of photoaging (**b**), LaSiOSi_PDMS before photoaging (**c**) LaSiOSi_PDMS after 400 h of photoaging (**d**), LaSi_PDMS before photoaging (**e**) and LaSi_PDMS after 400 h of photoaging (**f**,**g**).

The local nanomechanical characteristics were obtained from several force curves for each sample, which depict the cantilever’s deflection (DFL signal) as a function of the tip-sample distance measured during tip approach and retraction. Consequently, they are often referred to as the DFL-height curves. The DFL-height curves were obtained by AFM Force Curve Spectroscopy (FCS) in contact mode. The adhesion force was derived from the retract force curve using Hooke’s Law, accounting for the cantilever’s force constant and its displacement relative to the sample, based on the maximum pulling force at the final contact state before the tip’s detachment during withdrawal. In Figure 8, the representative approach (red) and retract (blue) DFL-height curves recorded for PDMS (a,b), LaSiOSi_PDMS (c,d) and LaSi_PDMS (e–g) before (a,c,e) and after 400 h of photoaging (b,d,f,g). Analyzing the DFL-height curves recorded for PDMS (Figure 8a) it can be observed that the sample has a viscoelastic behavior, typical for such polymers, suggested by the fact that when the material is pressed by the cantilever tip with a radius of curvature of 10 nm, it can withstand significant compression without damage, i.e., the material deforms reversibly. A mechanical hysteresis between the approach and retract curves is also observed, which is typical of soft and viscoelastic materials such as PDMS.

Photoaging of the PDMS sample for 400 h stiffened the material from a nanomechanical perspective, as indicated by the DFL-height curves in Figure 8b. This effect is likely attributable to a crosslinking phenomenon that produced a denser structure, reduced elasticity, and increased brittleness. The hysteresis between the approach and retract curves in this instance is minimal, further demonstrating reduced viscoelastic behavior, since the material is no longer capable of dissipating energy as effectively as the unmodified PDMS sample. In this case, the average adhesion force derived from the retraction curve was 225 ± 20 nN.

For the LaSiOSi_PDMS composite (Figure 8c) the slope of the approach curve is steeper than that obtained for the neat PDMS case, indicating a moderate increase in stiffness due to the introduction of the LaSiOSi complex into the PDMS matrix. The retract curve is slightly shifted relative to the approach curve, indicating energy loss (hysteresis), indicating that the newly created material still exhibits viscoelastic characteristics. At the same time, a slight plastic deformation or slow relaxation of the material can also be observed, resulting from the network formed in the composite. Following 400 h of photoaging of the LaSiOSi_PDMS sample (Figure 8d), the slope of the DFL-height curve is initially steep, suggesting that additional crosslinking or controlled chemical degradation reduced chain flexibility, thereby increasing rigidity. The tip no longer penetrates the material significantly. The approach and retract curves are almost superimposed, with hysteresis almost nonexistent, clearly indicating a decrease in viscoelasticity. The mean adhesion force was 356 ± 13 nN, higher than the one calculated for the PDMS sample subjected to photoaging for 400 h.

The integration of the LaSi complex into the PDMS matrix resulted in a very steep approach curve (Figure 8e), but not as pronounced as that seen in the LaSiOSi_PDMS composite. This indicates a small improvement in stiffness, less than that achieved for the LaSiOSi_PDMS film, likely because the Si-O-Si bond provides an additional network structure that is absent here. The significant disparity between the approach and retract curves indicates pronounced hysteresis and viscoelastic behavior; the sample exhibits increased stiffness while largely preserving its functional elasticity compared with the unmodified PDMS sample. Conversely, the LaSi complex appears to increase surface contact, as the tip appears slightly restrained by the sample. After 400 h of photoaging, the LaSi_PDMS sample exhibits two subtly distinct behaviors on the examined surface. The typical DFL-height curves for these two situations are shown in Figure 8f,g. Both curves initially exhibit a pronounced slope, indicating increased stiffness subsequent to the photoaging process. The material exhibits little deformation, and the tip penetrates minimally, corroborating its glassy nature. The absence of hysteresis between the approach and retract curves indicates that the material has lost significant viscoelastic characteristics. Nonetheless, the DFL-height curves, shown in Figure 8f, are recorded in homogeneous areas of the sample, demonstrating the characteristic behavior of a rigid, cross-linked, and uniform material, with a low-to-medium adhesion of 70 ± 5 nN. Conversely, the DFL-height curves akin to that depicted in Figure 8g exhibit an anomalous segment at the onset of the retract curve (a pinched or zig-zag region), succeeded by a pronounced local adhesion (193 ± 7 nN) where the tip seems to become ensnared in the material and is subsequently released abruptly, indicating substantial surface interactions. This local non-uniformity may be ascribed to an area rich in the LaSi complex.

### 3.4. Proposed Photoaging Mechanism

The FTIR analysis showed that the greatest reduction of infrared signals appeared for the neat PDMS, the highest hydrophily and development of –Si-O-Si- linkages and SiO_x_ structures was observed in LaSi_PDMS. The matrix in LaSiOSi_PDMS was quite resistant to UV-aging, and the levels of hydroperoxides and silanols were lower than in the other two films. Fluctuations in the water contact angle can be explained by two concurrent processes: thermally stimulated hydrophobic recovery of the PDMS matrix and occasional washing out of low-molecular mass photoproducts, thereby exposing less-damaged regions of PDMS from beneath.

**Scheme 2:**
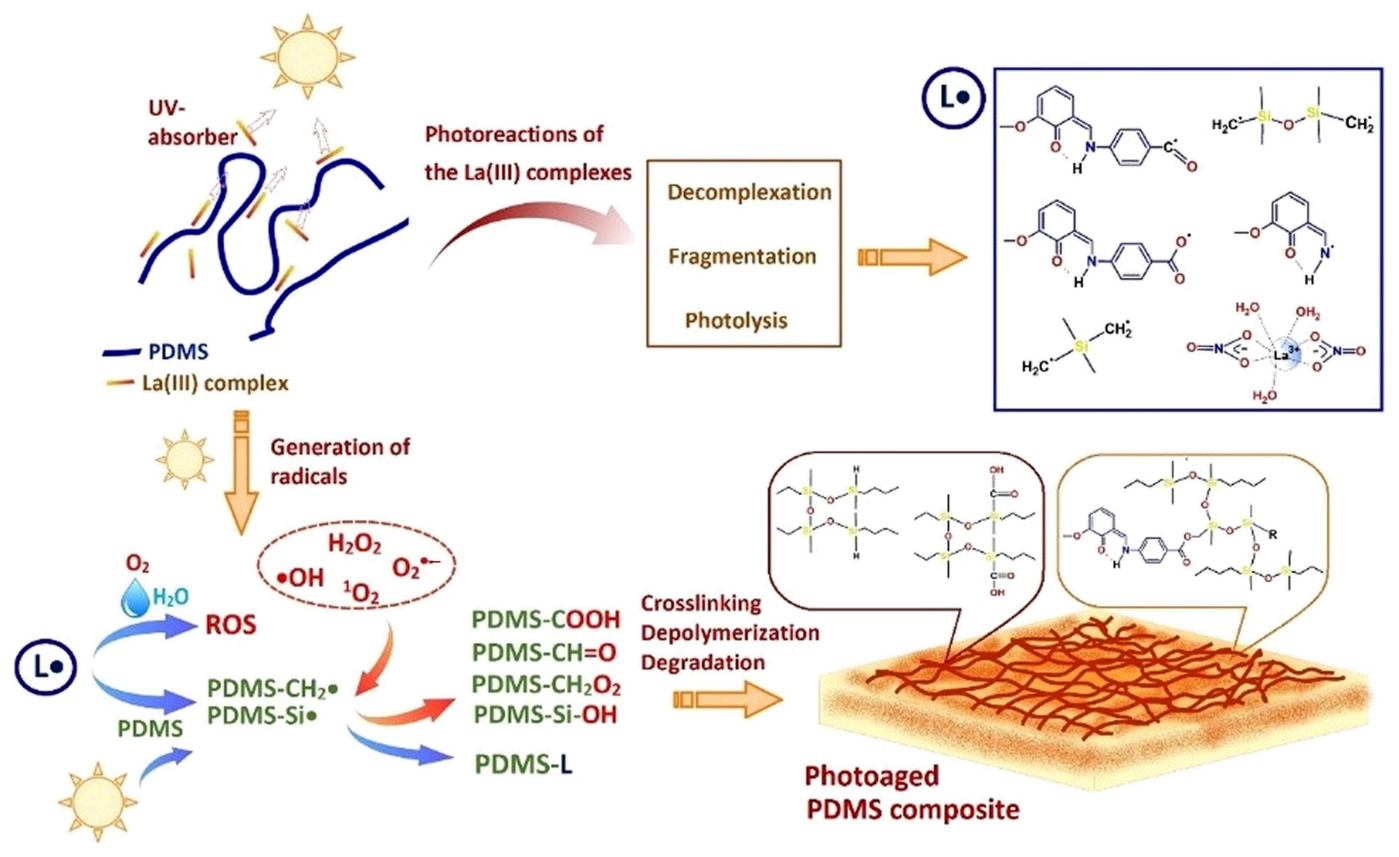
Proposed photoaging mechanism of the surface of composite PDMS films.

Based on the above analyses, the photoaging mechanism of composites is presented in Scheme 2. Photodegradation of neat PDMS film follows the photochemistry in the presence of atmospheric O_2_ [17,18,19]. In the presence of La(III) complexes, the photooxidation of PDMS has been replaced in the first stages by photoreactions of the fillers, followed by the interaction between the photoproducts and the PDMS matrix. The photoaging chamber contains O_2_ and atmospheric water vapors (RH of 60%). The interaction of the ligands, as radicals or excited species, and of PDMS with this atmosphere generates reactive oxygen species (ROS, OH•, H_2_O_2_, ^1^O_2_, and O_2_^•−^), as observed for many other polymeric hosts [27,28,29,30]. Also, PDMS is highly permeable to molecular oxygen [59], thereby enhancing surface photooxidation. The ROSs oxidize PDMS and the fragmented ligands, leading to scission on both sides and main chains, and attack the photoproducts, generating more radicals.

**Scheme 3:**
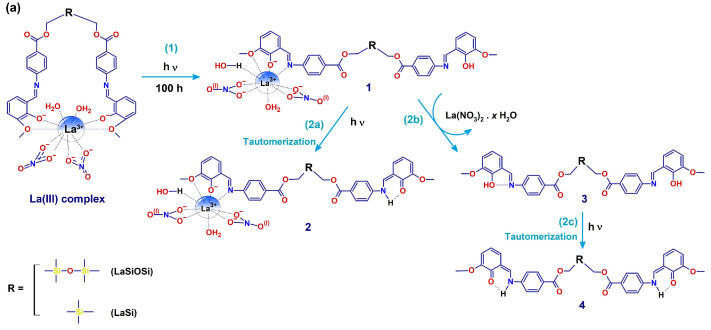

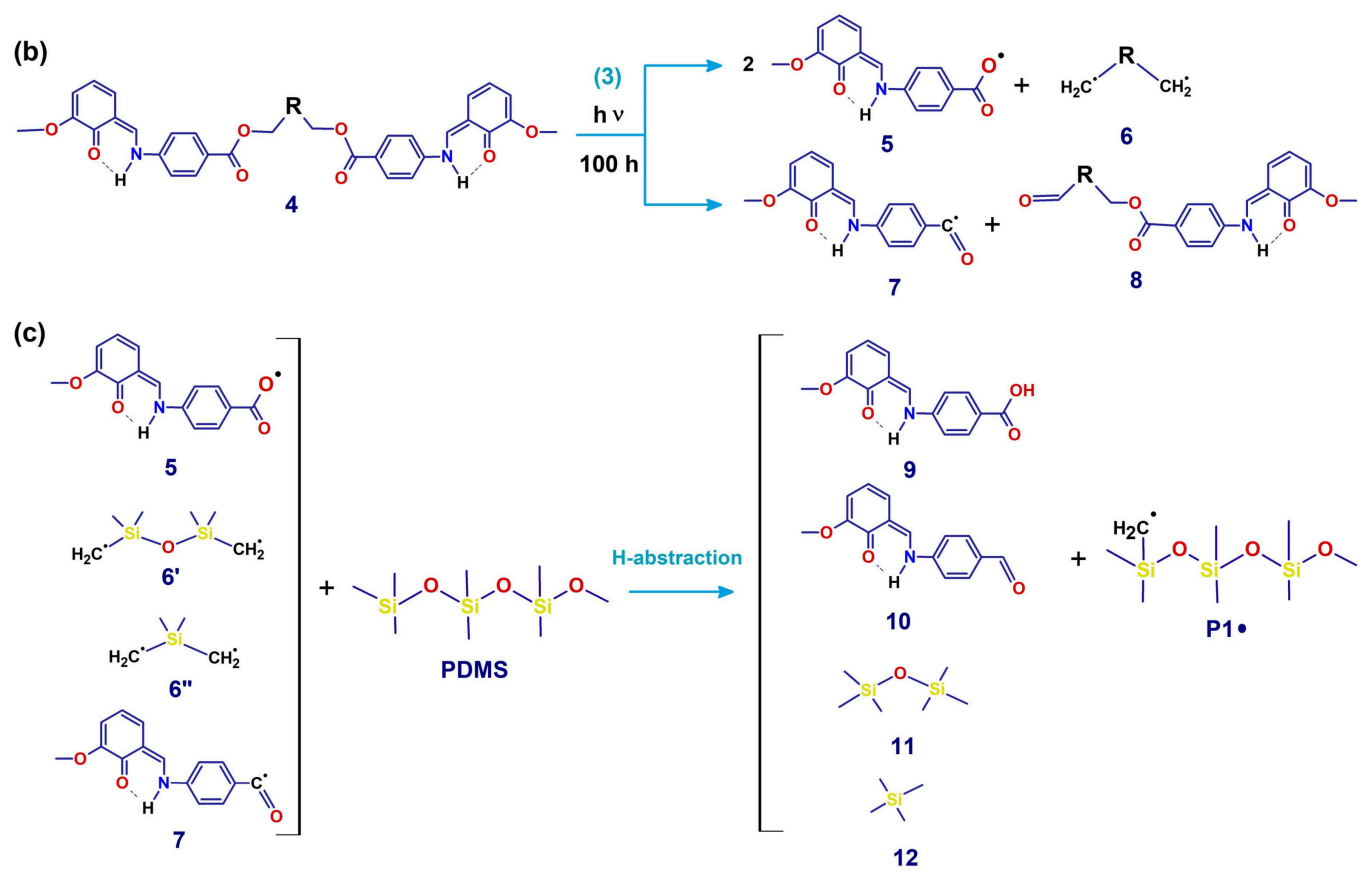
The first stage of the photoreaction chains on the surface of PDMS composites, taking place in the first hours of UV exposure: (**a**) dissociation of La(III) complexes and tautomerization of the uncomplexed segment of the ligand; (**b**) photolysis of dissociated ligands; (**c**) H-abstraction from the surrounding PDMS chains by the radicals derived from the ligands.

The primary photochemical reaction at the surface was the absorption of UV light by the La(III) complexes (Scheme 3), triggering a cascade of events: complex dissociation, tautomerization, photodegradation, and photolysis of the ligands. Fragmentation of the LaSi complex and detachment of the silane unit may happen because there is no Si-O bond in the spacer, and it is easier to break a Si-C bond (energy of 3.29 eV) than an Si–O bond (4.68 eV) [60]. In the Schiff base functionality, enol-to-keto tautomerism occurs (reactions (2a) and (2c)) [34,35] as shown by the FTIR spectral analysis (Figure 1). The aromatic and (semi)quinoid rings in the enol and keto tautomers are very good UV-absorbers, and excitation into the triplet state will convert them into highly reactive species [61,62,63].

All samples developed carbonyl-containing groups, especially hydroperoxides, acids, and ketones/aldehydes, indicating that the PDMS matrix was mainly (photo)oxidized. In these processes, PDMS acted as a scavenger of ROS. The highest rates of photooxidation and photodegradation of the PDMS matrix were observed in LaSi_PDMS, occurring when the ligand was photolyzed, and small radicals initiated an H-abstraction chain reaction.

The carbonyl-containing groups can result from several photoreactions: (i) photolysis of partially dissociated species **1** and its tautomeric form **2**, resulting in either the ligand (**3**) or its quinoid form **4**; (ii) according to the photochemistry of benzoates [64,65] the α- or β- cleavage or **3**/**4** results in benzoyloxy (**5**) or benzoyl radicals (**6**), and in detachment of the silane/siloxane segment. The radicals produced by photolysis act as initiators of free-radical chain reactions and photooxidation reactions when excited in the singlet or triplet state. Hydrogen abstraction from PDMS methyl groups results in the propagating macroradical P1• in Scheme 3c, and several low-molar mass compounds (benzoic acid derivative **9**, benzaldehyde derivative **10**, hexamethyl disiloxane **11,** and tetramethylsilane **12**). The carbonyl compounds such as **9** and **10** are highly photosensitive and prone to further photoreactions, as Chen et al. [61], He et al. [62] and Wan et al. [63] have shown.

**Scheme 4:**
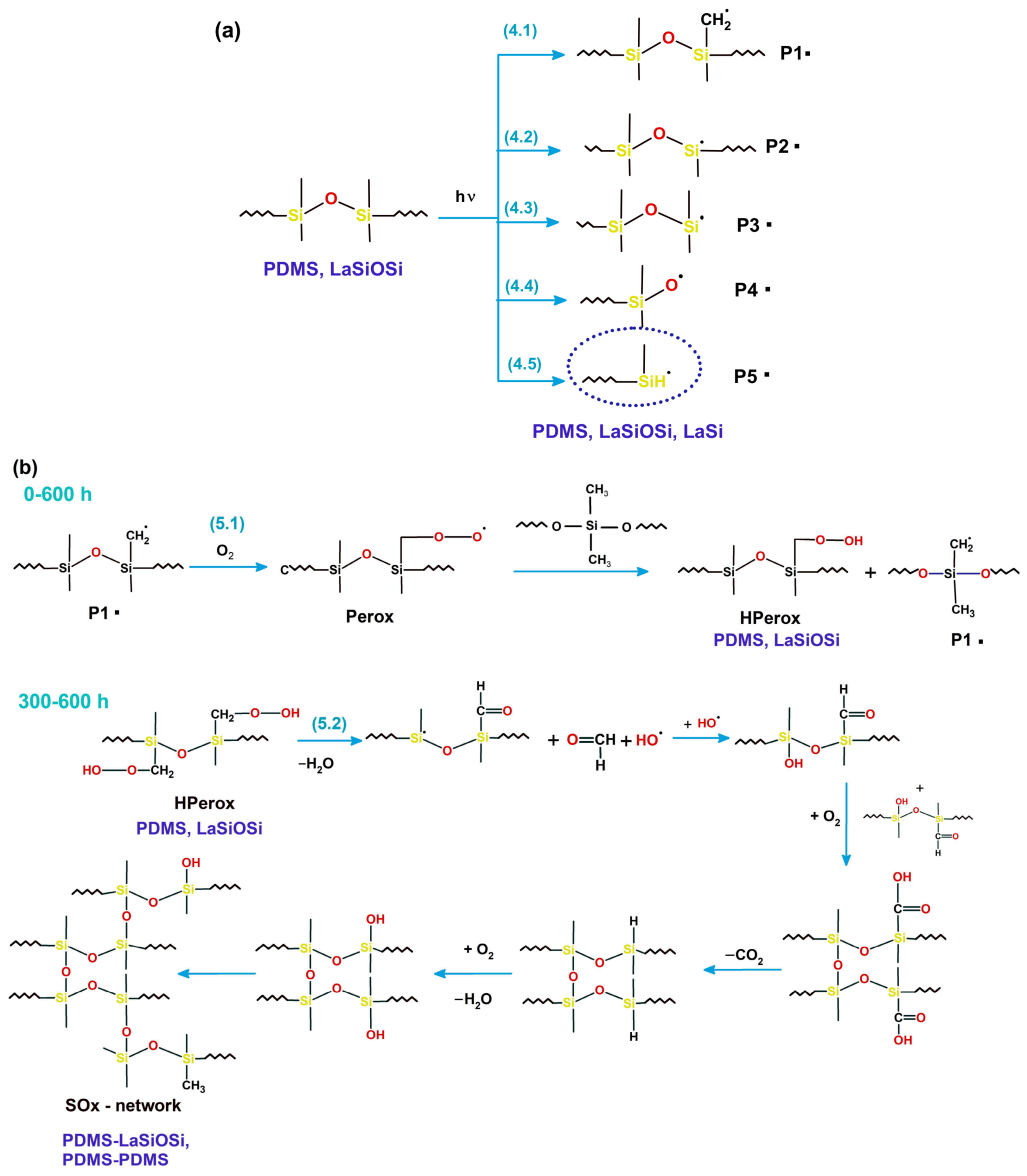
Photoreactions in the siloxane segment of PDMS and of the LaSiOSi ligand: (**a**) the five potential radicals obtained at the siloxane site; (**b**) Generation of the SiOx network initiated by the decomposition of hydroperoxides.

The photoreactions of the siloxane segment in the SiOSi-ligand with PDMS are identical to those taking place within the native PDMS [17,18,19,66,67]. Five kinds of macroradicals can potentially be generated by UV photons for long irradiation periods, as shown by reactions (4.1)–(4.5) in Scheme 4a. P1• appears by hydrogen abstraction, P2• by side-chain scission, and P3•–P5• appear by the breaking of the main chain [17,18,19,66,67]. Crosslinked structures based on SiO_x_ bonds of various Si-O-Si angles occur by a chain of reactions. The first step is the decomposition of the hydroperoxide groups (Scheme 4b), and the final stage is the condensation of silanol units with water elimination, at advanced photoaging times. The silane/siloxane spacers from the ligands participate in the SiO_x_ structures according to the crosslinking reactions of silicones (Scheme 4b) [17,18,19,45,66,67]. The final result is an extended SiO_x_ network that locally incorporates ligand fragments as crosslinking bridges.

The degradation rate was estimated from the DFL-height curves (AFM technique), by analyzing the nanomechanical characteristics of the surface and the adhesion forces. We determined that the degradation rate for PDMS alone was 0.56 nN/h, for LaSiOSi_PDMS was 0.89 nN/h, and for LaSi_PDMS was 0.48 nN/h.

**Scheme 5:**
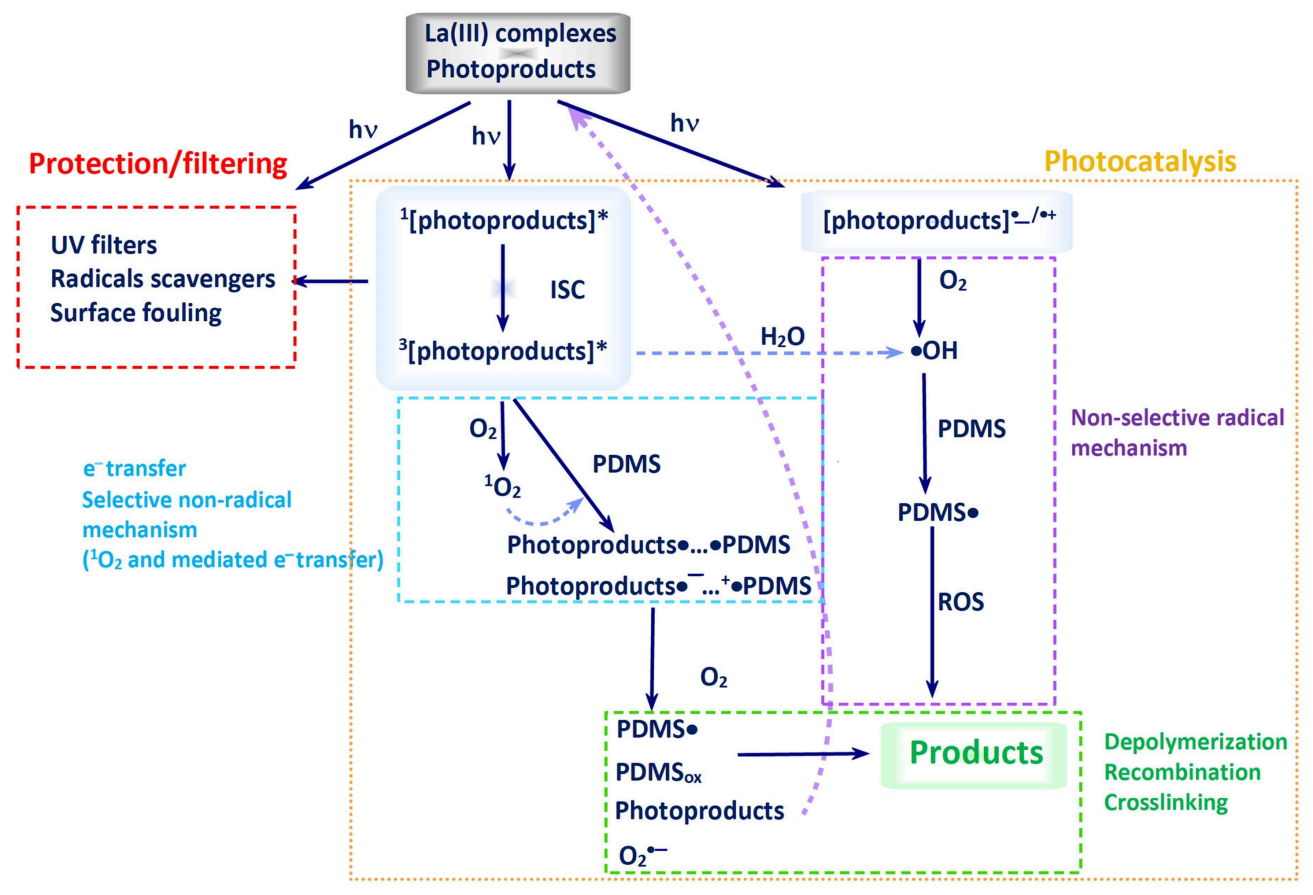
The role of La(III) complexes in the photoinitiated degradation of PDMS during photoaging. ISC = intersystem crossing from the first singlet excited state to the triplet state of the photoproducts.

Photoproducts, recombination products, and the radical intermediates interfere with the degradation mechanism, especially when deposited on the film surface. These species can inhibit the photodegradation of PDMS by playing protective roles as UV filters for the bulk PDMS composite (since neat PDMS is UV-transparent), as oxygen and radical scavengers, or by producing the surface fouling and inhibiting the generation of reactive species. The result can be observed as a cyclic and in tandem variation of WCA, adhesion forces values, and ESR signals (Figure 6, Figure 7 and Figure 8)). Summing up all these observations, Scheme 5 proposes the processes induced by the La(III) complexes, their ligands, and the photoproducts.

### 3.5. Hydrophobic Recovery

The PDMS surface treated with UV/ozone, plasma, or high–energy partially recovers its hydrophobic properties over time, driven by the low surface energy of oxidized PDMS chains. The pre-existing oligomers in PDMS or in situ generated species migrate toward the surface and replace the SiO_2_–carrying chains through cracks or the microporous structure of the crosslinked layer [68,69]. The surface chemistry of photoaged PDMS composites was periodically monitored by ATR-FTIR spectroscopy, after storage in sealed boxes in the dark.

**Figure 9:**
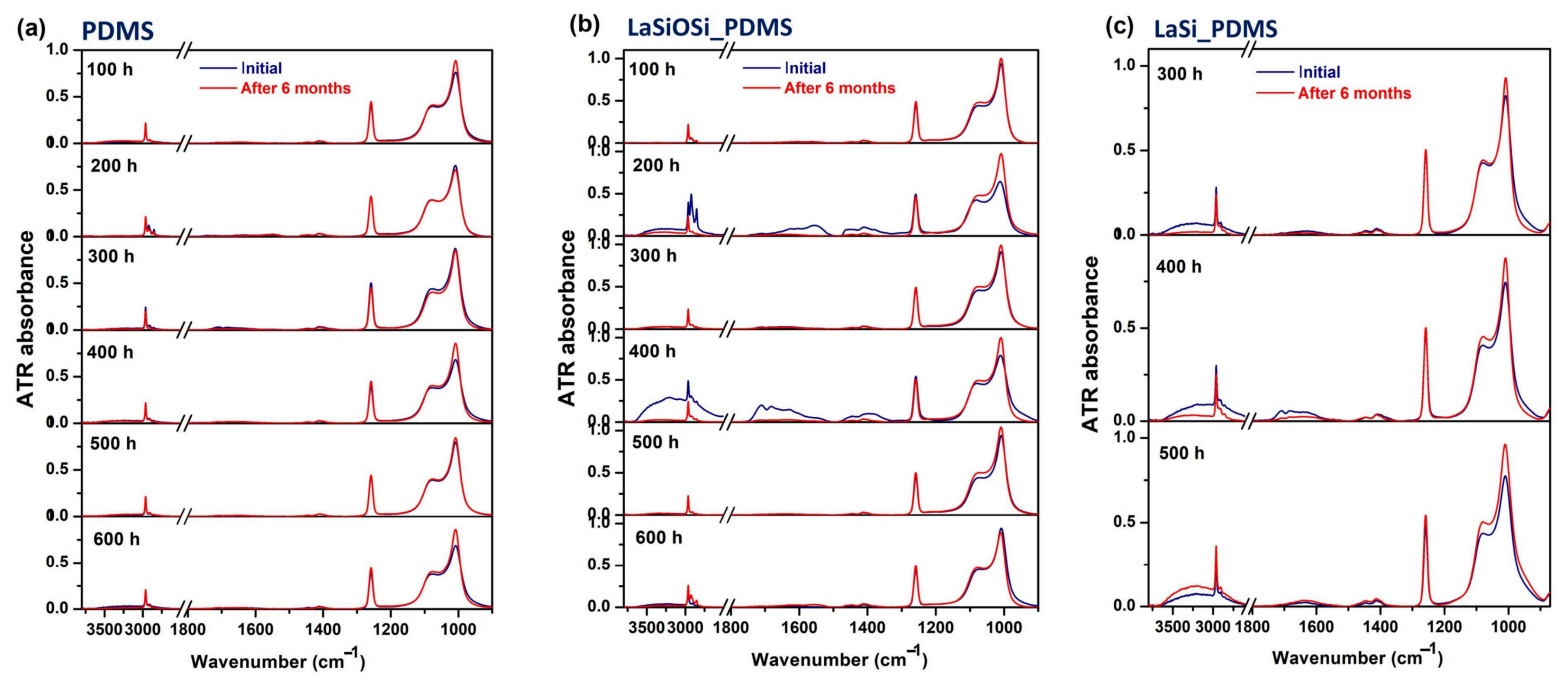
Hydrophobic recovery of the surface of photoaged PDMS composites after 6 months of storage in the dark, analyzed by means of ATR-FTIR spectroscopy: (**a**) PDMS; (**b**) LaSiOSi_PDMS; (**c**) LaSi_PDMS.

Figure 9 presents the ATR-FTIR spectra after 6 months of storage, which corresponds to the equilibrium state and the final structure of the top layer. All samples show amelioration, but not disappearance, of OH/silanols/hydroperoxide signals, and a clear increase in the intensities of methyl and Si-O-Si vibrations at any photoaging time. LaSi_PDMS still has some adsorbed water, hydroperoxide, and/or free silanol groups on its surface, but after longer aging times, it appears that degradation still continued in the dark. The slow recovery of LaSi_PDMS may be affected by several factors: (i) the H-bonding of the surface silanol/hydroperoxide groups with photoproducts chemisorbed on the surface and/or with the adsorbed water; (ii) the inability of polar groups from the surface to reorient towards bulk, probably related to the mesh size and crosslinking density of the silica-LaSi network; (iii) the mesh size, the rigidity and thickness of the silica-LaSi network that controls the migration of low molecular compounds from the bulk to the surface; (iv) a continuous electron transfer from reactive photoproducts to PDMS and between photoproducts; (v) the high reactivity of aromatic and quinone groups of the photoproducts in dark conditions, by activating surrounding species with subsequent generation of •OH radicals [29,30]. Whatever the extent of chain cleavage and recombination, the network having fragments of La(III) complexes as crosslink points is firm and strong. This barrier hindered the molecular diffusion of hydrophobic low-molecular weight compounds (LMW) and reduced the matrix’s healing ability.

Our findings provide a new perspective on the behavior of PDMS under simulated solar conditions after catalysis by organometallics whose chemical structures are partially similar to that of the matrix and contain several reactive units. Following well-defined procedures for crosslinking and well-starting compounds: PDMS of known molar mass, viscosity, etc., the amount of the crosslinker–TEOS and catalysts and metal complexes with defined structures, it can be considered that the process of production of PDMS hybrid films with La(III) complexes with a silicon-containing structure can address the problem of processability of metal–loading composites. Lastly, we note that the benzoate group has recently been promoted as an efficient photosensitization catalyst by Yakubov et al., who used methyl 4-fluorobenzoate for a regioselective fluorination of a C(sp^3^)–H bond [70]. This research highlights the previously unexplored potential of photosensitive benzoates as a next-generation photocatalyst.

## 4. Conclusions

In this work, we analyzed the structural and chemical changes induced by photoaging on the surfaces of two La(III) complexes-PDMS composites, compared with undoped PDMS. The fillers act as photocatalysts of PDMS degradation. (Photo)oxidation of the PDMS matrix was observed using ATR-FTIR and UV-Vis spectroscopy, as evidenced by the development of carbonyl-containing groups (hydroperoxides, acids, and ketones/aldehydes). A net difference in the surface morphology between the two photoaged PDMS composites was found, as the oxidized superficial layer had a sea-island appearance in one composite and a dimple pattern in the other. Both photoaged composites are located in the hydrophobic zone based on water contact angle values. The adhesion forces of LaSiOSi_PDMS are higher than those of PDMS and LaSI_PDMS and can be attributed to photoproducts spread on the surface. The degree of hydrophobic recovery of composites after several months is small, suggesting that the healing of the PDMS matrix was impeded by the stiffness of the crosslinked superficial layer, which “froze” existing cracks and blocked diffusion of PDMS chains from beneath. An in-depth analysis of the role of fillers in the photoinitiated degradation of PDMS allowed us to propose the photoaging mechanism.

## Figures and Tables

**Table 1:** Data extracted from the TG and DTG curves.

Sample	Degradation Stage	*T*_5%_ °C	*T*_peak_ °C	*W* %	*T*_30_ °C	*T*_s_ °C	*T*_50_ °C	*R* %
PDMS	I	363	387	20.78				
	II		424	30.89	422	195	466	
	III		519	25.03				
	IV		671	2.21				20.09
PDMS–600 h	I	441	497	72.16	501	234	534	
	II		661	8.90				17.88
LaSi_PDMS	I	327	344	7.05				
	II		446	38.39	442	194	498	
	III		539	27.75				
	IV		670	3.02				23.27
LaSi_PDMS–600 h	I	415	437	44.65				
	II		637	27.49	555	245	639	27.6
LaSiOSi_PDMS	I	322	345	30.6				
	II		455	28.96	392	178	451	
	III		494	18.77				21.11
LaSiOSi_PDMS–600 h	I	411	445	35.05				
	II		527	37.88	463	217	501	26.54

*T*_5%_—temperature corresponding to 5 wt.%, mass loss; *T*_peak_—temperature corresponding to the maximum rate of decomposition; *T*_30_, *T*_50_—the temperatures corresponding to 30 wt.% and 50 wt.% mass losses; *W*—percentage of mass loss corresponding to each stage; *T*_s_—static heat resistance index; *R*—mass residue at 700 °C.

## Data Availability

The data will be made available upon request.

## Supplementary Materials

The following supporting information can be downloaded at: https://www.mdpi.com/article/10.3390/polym18111334/s1: Figure S1. IR spectrum of H_2_**L1**; Figure S2. IR spectrum of H_2_**L2**; Figure S3. ^1^H NMR spectrum of H_2_**L1**; Figure S4. ^13^C NMR spectrum of H_2_**L1**; Figure S5. ^1^H NMR spectrum of H_2_**L2**; Figure S6. ^13^C NMR spectrum of H_2_**L2**; Figure S7. IR spectrum of [La**L1**(NO_3_)_2_]; Figure S8. IR spectrum of [La**L2**(NO_3_)_2_(H_2_O)]; Figure S9. ^1^H NMR spectrum of [La**L1**(NO_3_)_2_]; Figure S10. ^1^H NMR spectrum of [La**L2**(NO_3_)_2_(H_2_O)]; Figure S11. MALDI-TOF/ TOF-MS spectrum of [La**L1**(NO_3_)_2_]; Figure S12. MALDI-TOF/ TOF-MS spectrum of [La**L2**(NO_3_)_2_H_2_O]; Figure S13. SEM Cross-section in the LaSiOSi_PDMS films at different depths and EDX composition of the films; Figure S14. SEM Cross-section in the LaSi_PDMS films at different depths and EDX composition of the films; Figure S15. SEM image of the surface of LaSi_PDMS film and the EDX composition in the selected region; Figure S16. SEM image of the surface of LaSiOSi_PDMS film and the EDX composition in the selected region; Figure S17. TG and DTG curves of initial samples and photo–aged for 600 h; Figure S18. DSC thermograms of photoaged films: (a) native PDMSD; (b) LaSiOSi_PDMS; (c) LaSi_PDMS; Figure S19. Evolution of infrared spectra during photoaging for: (a) neat PDMS (b) LaSiOSi_PDMS; (c) LaSi_PDMS; Figure S20. Effects of accelerated UV-aging on the fluorescence of LaSi/LaSiOSi_PDMS films compared to the powder form of pure complexes (dark blue line): (a) LaSiOSi_PDMS; (b) LaSi_PDMS; Table S1. Data extracted from the DSC curves of the studied sample.
