# Supplementary material for: Polydimethylsiloxane-Based Composites with Photo-Autocatalytic Properties: Surface Photooxidation, Hydrophobicity, and Nanomechanical Properties
Source: Polymers (Basel). 2026 May 28;18(11):1334. doi: 10.3390/polym18111334 (PMC13259371; doi:10.3390/polym18111334)
Supplement: Supplementary file 1 [file polymers-18-01334-s001.zip › polymers-4316343-supplementary.pdf]

Supporting Information

Polydimethylsiloxane – based composites with photo-autocatalytic properties: surface photooxidation, hydrophobicity, and nanomechanical properties

Mihaela Iuliana Avadanei[1,] Mirela-Fernanda Zaltariov,[1] Iuliana Stoica,[1] Cristian-Dragos Varganici,[1] Diana Elena Ciolacu,[1] Iuliana Spiridon,[1] Adrian Fifere,[2] Ovidiu Gabriel Avadanei[3,]**

[1] Petru Poni" Institute of Macromolecular Chemistry, 41A Gr. Ghica Voda Alley, 700487, Iasi ROMANIA
[2]Centre of Advanced Research in Bionanoconjugates and Biopolymers, "Petru Poni" Institute of Macromolecular Chemistry, 700487 Iasi, Romania
[3]Faculty of Physics, "Alexandru Ioan Cuza" University,11 Carol Blvd., 700506 Iasi, ROMANIA

Table of contents

S1. Synthesis of ligands and of La(III) complexes

S2. Structural analysis of ligands and La(III) complexes

S3. Thermal analysis of photoaged La(III) complexes – PDMS composites

S4. ATR-FTIR and fluorescence spectra of photoaged PDMS-based composites

S1. Synthesis of ligands and of La(III) complexes

The La(III) complexes have been synthesized in two steps: (1)- the preparation of the Schiff base ligands starting from 1,1,3,3-(tetramethyldisiloxane-1,3-diyl)-bis-(methylene)-bis-(*p*-aminobenzoate) (0.216 g, 0.5 mmol) or dimetilsilan-bis-(metilen)-bis-(p-aminobenzoat) (0.179 g, 0.5 mmol) and o-vanillin (0.152 g, 1 mmol) dissolved in acetonitrile (10 mL, 1:1, v:v) and stirred to reflux for 2 h, followed by filtration, washing and drying of the precipitate at RT; (2) - the preparation of the La(III) complexes by mixing the Schiff base products isolated in the first step (0.14 g, 0.2 mmol) dissolved in methanol (10 mL) with La(III) nitrate (0.065 g, 0.2 mmol) in

methanol (5 mL). The resulting mixture was stirred at 50°C overnight. The precipitate formed was filtered off, washed with cold methanol and dried at RT.

(*E*)-(tetramethyl-disiloxanediyl)-bis(methylene)-bis(4-((*E*)-(2-hydroxy-3-methoxybenzilidene)-amino)benzoate (H_2**L1**) **(**$C_{36}H_{40}N_2O_9Si_2$) (0.3 g, yield 85.7%). ***IR*** ν_{max} (KBr pellet), cm^{-1}: 399w, 507vw, 550vw, 567w, 582w, 633w, 698m, 735s, 781s, 802s, 827s, 837s, 872m, 972m, 1016m, 1078vs, 1101s, 1173s, 1200m, 1252vs, 1279m, 1308s, 1364m, 1410m, 1441m, 1466s, 1504vw, 1576s, 1597s, 1618m, 1711vs, 2835w, 2897w, 2955w, 3072vw, 3402vw. ***^{1}H-NMR*** (400.13 MHz, $CDCl_3$, δ, ppm): 13.27 (s, 2H, OH), 8.59 (s, 2H, -CH=N-), 8.04 (d, 4H, Ar-H), 7.25 (d, 4H, Ar-H), 7.01-6.98 (t, 4H, Ar-H), 6.88-6.84 (t, 2H, Ar-H), 3.98 (s, 4H, Si-CH_2), 3.91 (s, 6H, Ar-CH_3), 0.233 (s, 12H, Si-CH_3). .***^{13}C-NMR*** (100.6 MHz, $CDCl_3$, δ, ppm): 166.61, 164.04, 152.01, 151.45, 148.44, 131.00, 130.88, 128.61, 124.00, 121.11, 118.85, 118.73, 115.22, 58.22, 56.14, 46.20, 11.56, -0.86.

(*E*)-(dimethylsilanediyl)-bis(methyl-ene)-bis(4-((*E*)-(2-hydroxy-3-methoxybenzilidene)-amino)-benzoate (H_2**L2**) **(**$C_{34}H_{34}N_2O_8Si$): **(**0.29 g, 90.6%). ***IR*** ν_{max} (KBr pellet), cm^{-1}: 569w, 611w, 636w, 698m, 735s, 777s, 806m, 851s, 972m, 1015m, 1101s, 1169s, 1200s, 1256vs, 1312s, 1367m, 1412m, 1464s, 1504w, 1576s, 1597vs, 1620s, 1651m, 1713vs, 2847w, 2924w, 2957w, 3061vw, 3439s. ***^{1}H-NMR*** (400.13 MHz, $CDCl_3$, δ, ppm): 13.31 (s, 2H, OH), 8.66 (s, 2H, -CH=N-), 8.10 (d, 4H, Ar-H), 7.31 (d, 4H, Ar-H), 7.07-7.04 (m, 4H, Ar-H), 6.92 (t, 2H, Ar-H), 4.25 (s, 4H, CH_2), 3.98 (s, 6H, Ar-CH_3), 0.36 (s, 6H, Si-CH_3). ***^{13}C-NMR*** (100.6 MHz, $CDCl_3$, δ, ppm): 166.67, 164.11, 152.12, 151.52, 148.49, 131.00, 128.47, 124.09, 121.14, 118.88, 118.79, 115.34, 56.23, -5.63.

[LaL1(NO$_3$)$_2$]- ($C_{36}H_{38}LaN_4O_{15}Si_2$) (0.11 g, yield 71.43%).%. Calculated for $C_{36}H_{38}LaN_4O_{15}Si_2$: C 44.96%, H 3.98%, N 5.83%. Found: 44.80%, H 3.63%, N 5.45%. ***IR*** ν_{max} (KBr pellet), cm^{-1}: 397w, 469w, 509m, 530w, 569m, 604w, 635w, 662m, 692m, 737s, 770m, 787m, 800m, 820m, 839m, 899w, 961w, 1013m, 1034m, 1067m, 1113m, 1169s, 1190m, 1200m, 1234s, 1254s, 1306vs, 1385vs, 1410s, 1456s, 1506s, 1547m, 1603vs, 1639vs, 1719s, 2839w, 2959w, 3435vs. ***^{1}H-NMR*** (400.13 MHz, DMSO$_{d6}$, δ, ppm): 12.72, 8.95, 8.02-7.98, 7.50-7.46, 7.27-7.24, 7.16-7.14, 6.94-6.89, 3.98, 3.82, 0.21. ***MALDI-TOF/TOF-MS*** m/z: for $C_{36}H_{38}LaN_4O_{15}Si_2$ $[M]^+$ calcd 961.7777, found 961.261.

[LaL2(NO$_3$)$_2$ (H$_2$O)]- ($C_{34}H_{34}LaN_4O_{15}Si$) (0.096 g, 68.5%). Calculated for $C_{36}H_{38}LaN_4O_{15}Si_2$: C 45.09%, H 3.78%, N 6.19%, Found C 44.86%, H 3.98%, N 5.98 ***IR*** ν_{max} (KBr pellet), cm^{-1}: 403w, 469w, 511w, 569w, 665vw, 687w, 746m, 791m, 822m, 851m, 883w, 964m, 1011m, 1034m, 1069w, 1115s, 1169s, 1240vs, 1300vs, 1366s, 1458vs, 1497vs, 1547m, 1603s, 1639vs, 1730s, 2843vw, 2918w, 3431m. ***^{1}H-NMR*** (400.13 MHz, $CDCl_3$, δ, ppm): 12.71, 8.93, 7.98-7.96, 7.45-7.43, 7.24-7.22, 7.16-7.14, 6.90-6.86, 4.18, 3.82, 0.26. ***MALDI-TOF/TOF-MS*** m/z: for $C_{34}H_{34}LaN_4O_{15}Si$ $[M+Na]^+$ calcd 928.63, found 928.15.

S2. Structural analysis of ligands and La(III) complexes

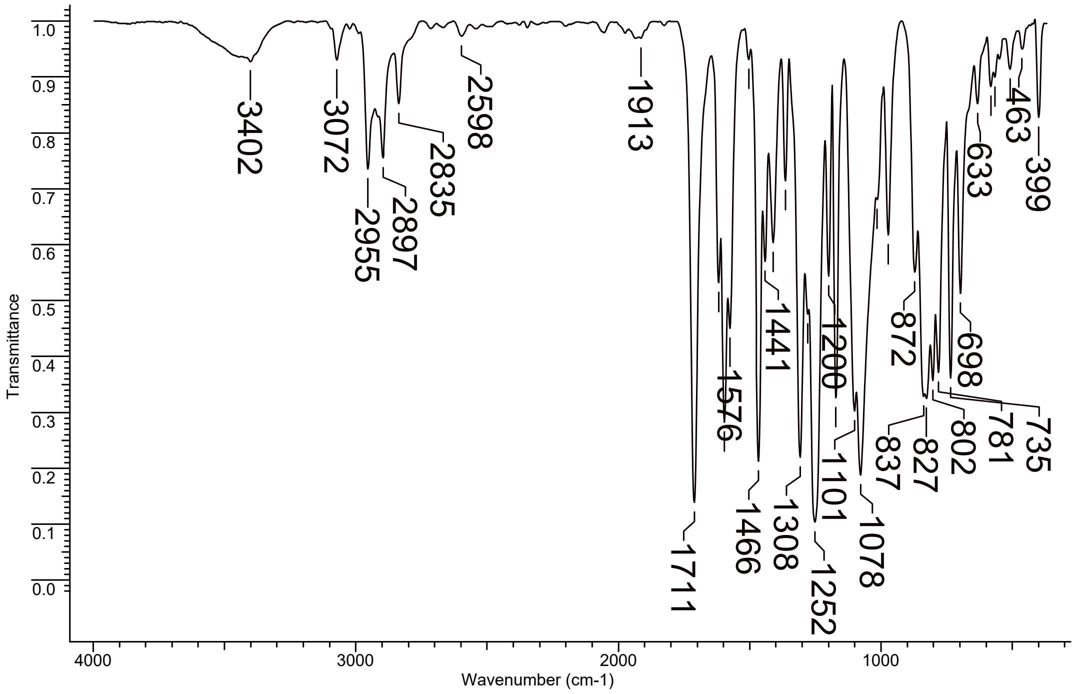

Figure S1. IR spectrum of H_2**L1**

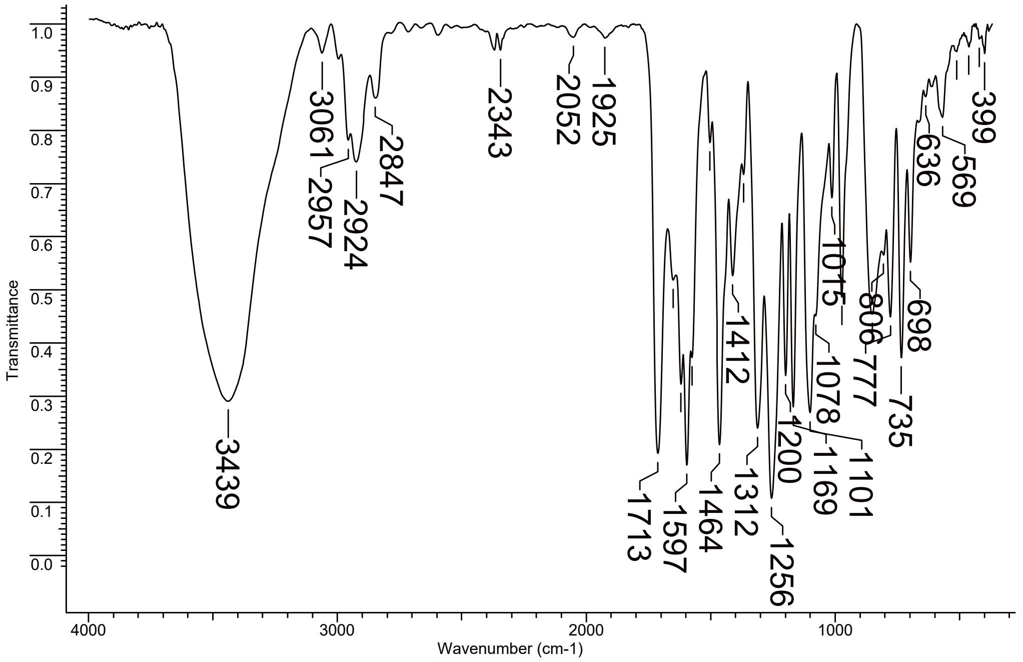

Figure S2. IR spectrum of H_2**L2**

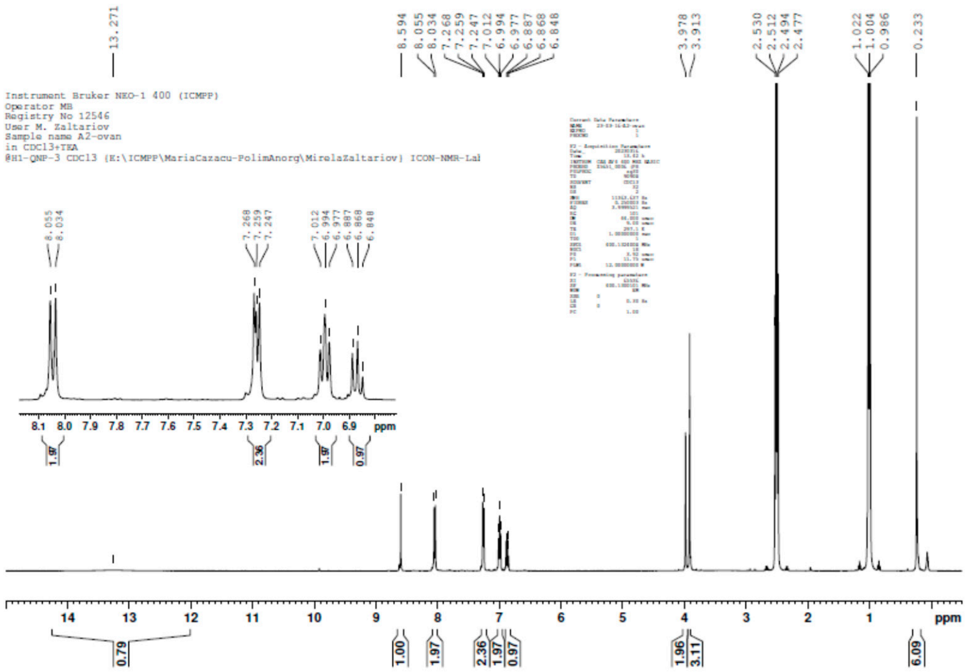

Figure S3. ^{1}H NMR spectrum of H_2**L1**

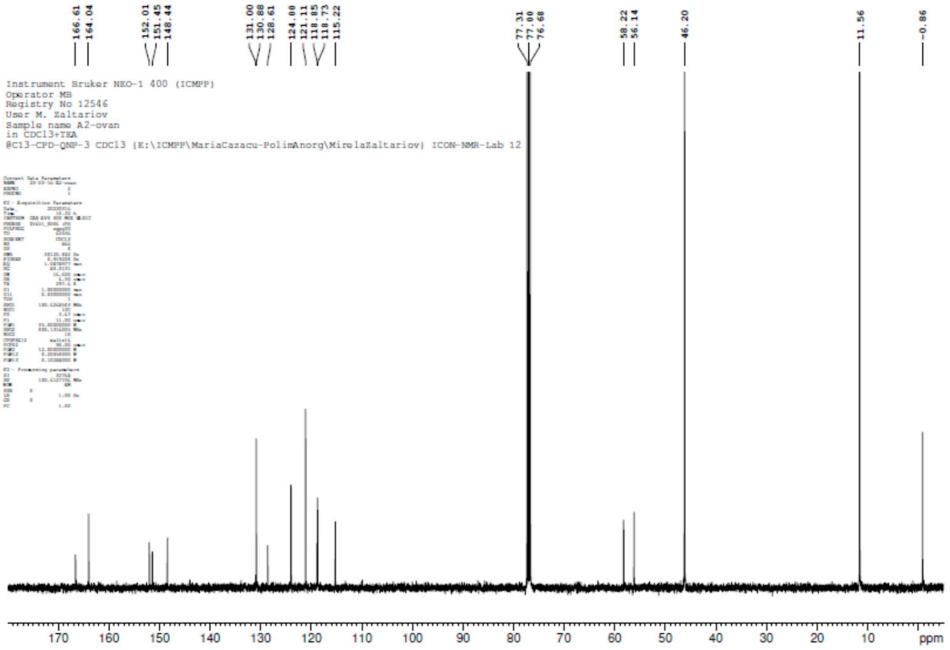

Figure S4. ^{13}C NMR spectrum of H_2**L1**

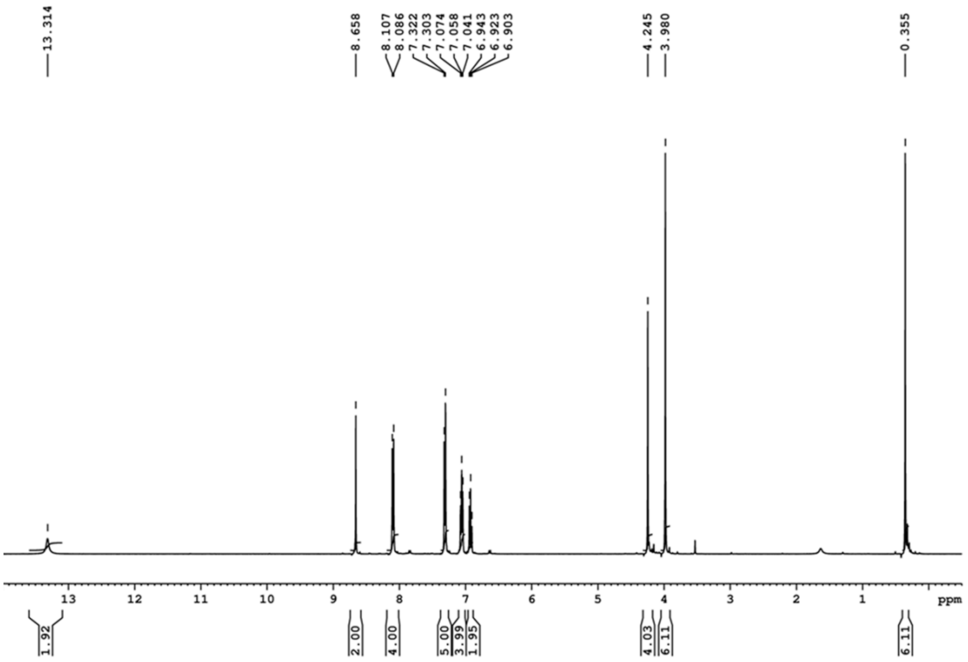

Figure S5. ^{1}H NMR spectrum of H_2**L2**

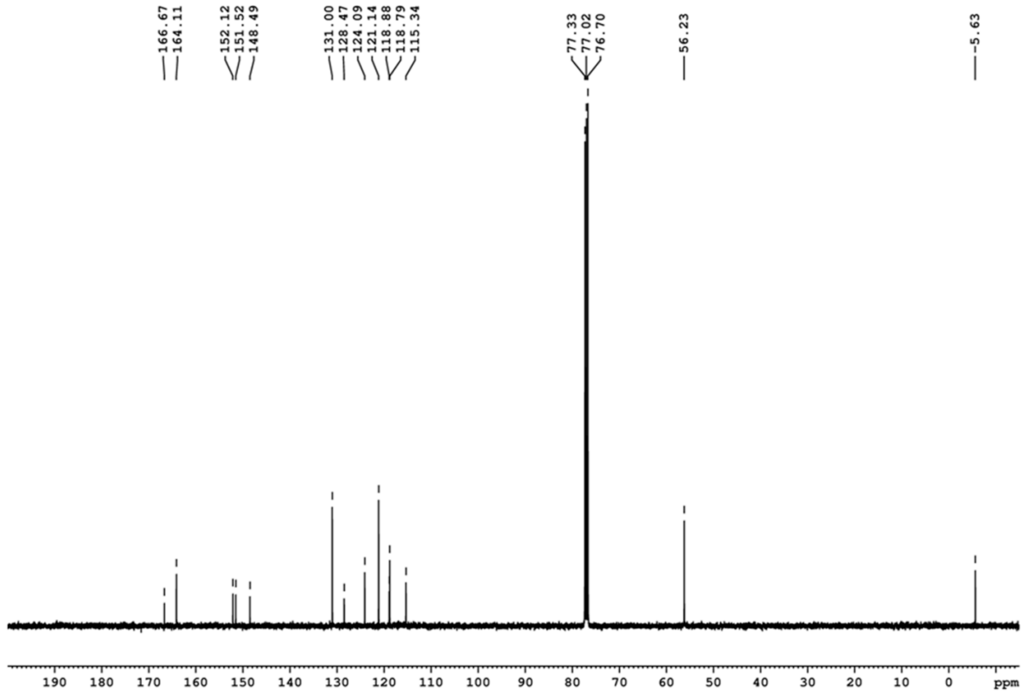

Figure S6. ^{13}C NMR spectrum of H_2**L2**

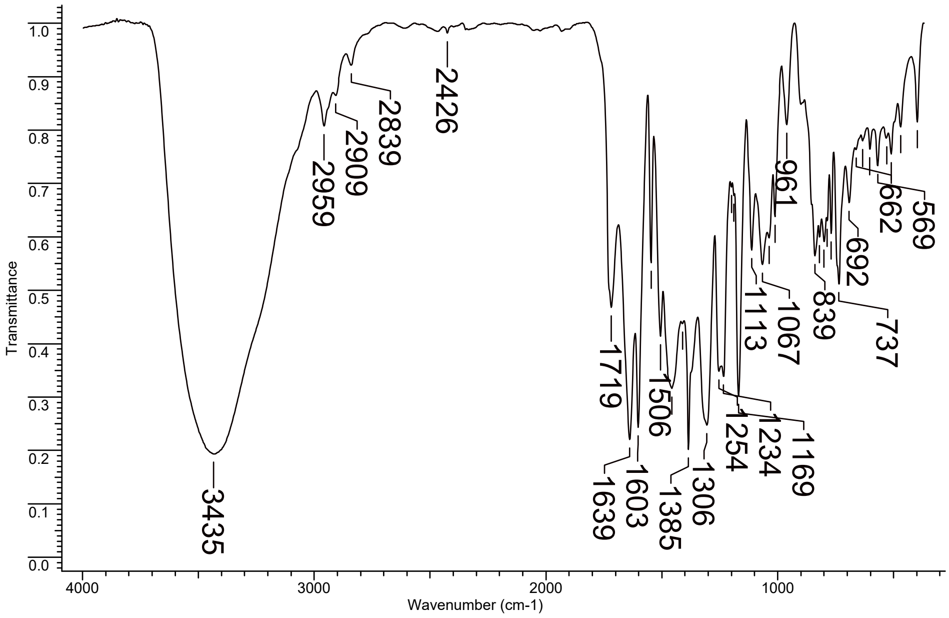

Figure S7. IR spectrum of [La**L1**(NO_3)$_2$]

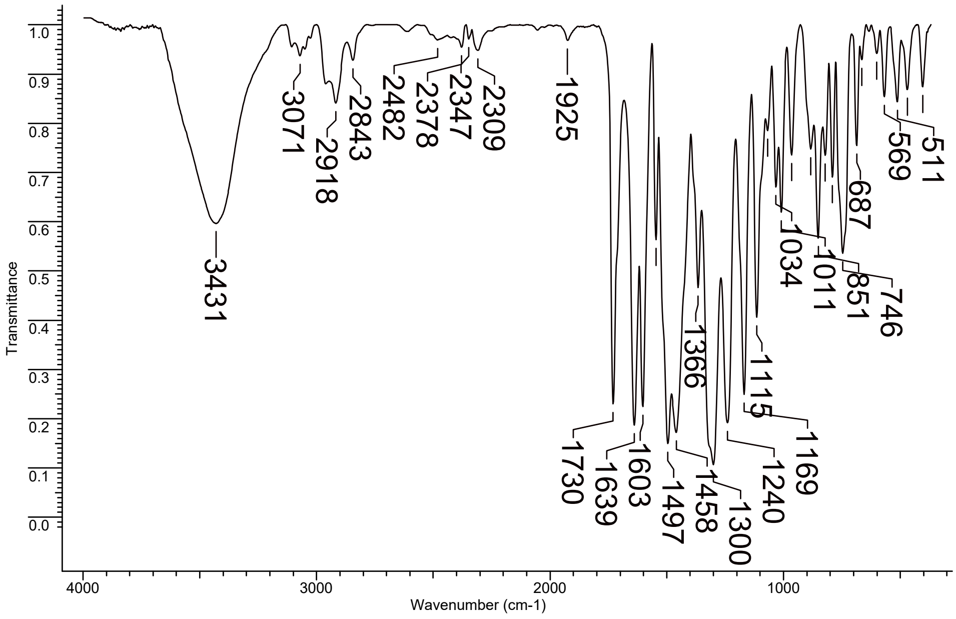

Figure S8. IR spectrum of [La**L2**(NO_3)$_2$(H_2O)]

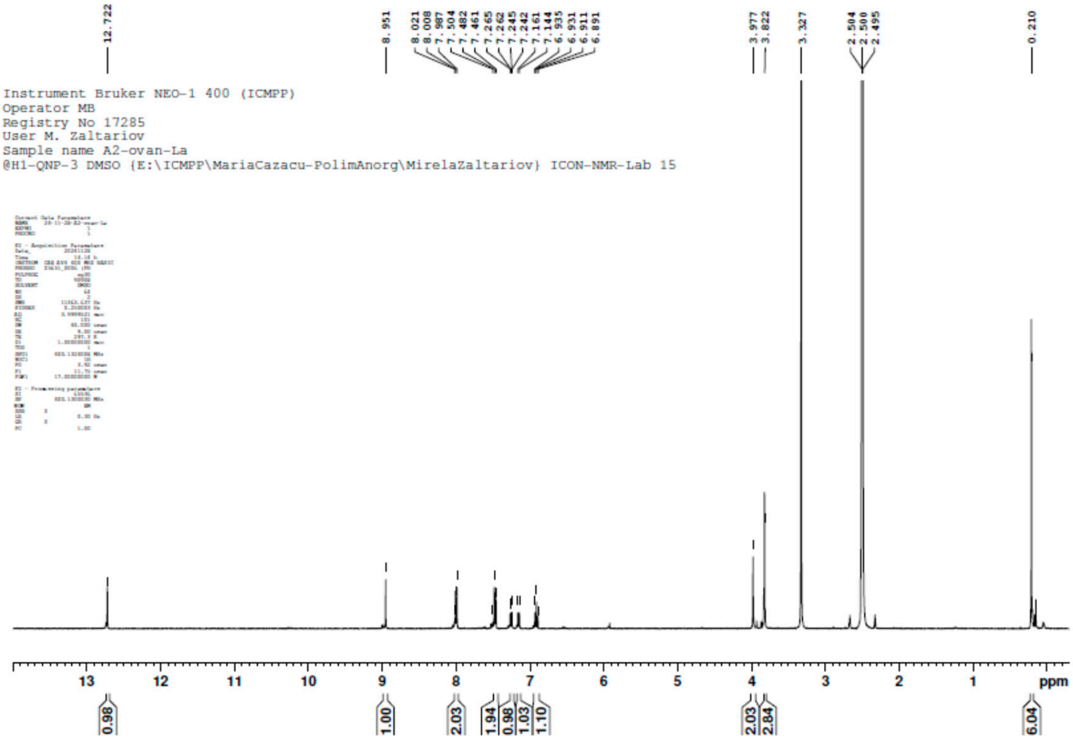

Figure S9. ^{1}H NMR spectrum of [La**L1**$(NO_3)_2$]

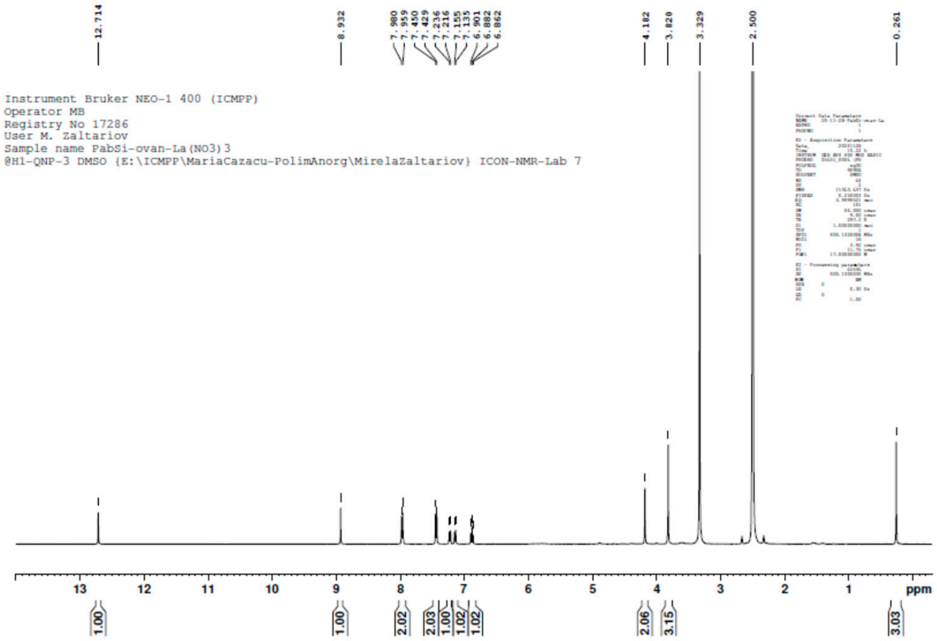

Figure S10.^{1}H NMR spectrum of [La**L2**$(NO_3)_2(H_2O)$]

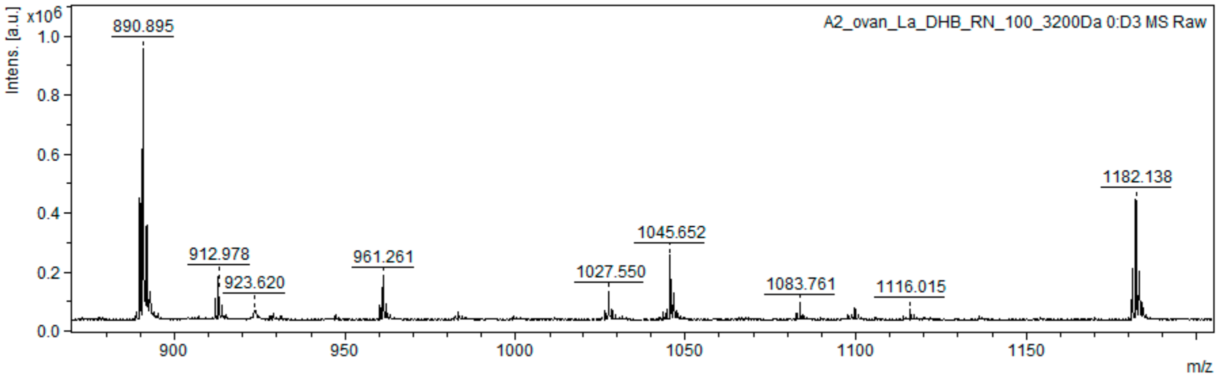

Figure S11. MALDI-TOF/ TOF-MS spectrum of [La**L1**$(NO_3)_2$]

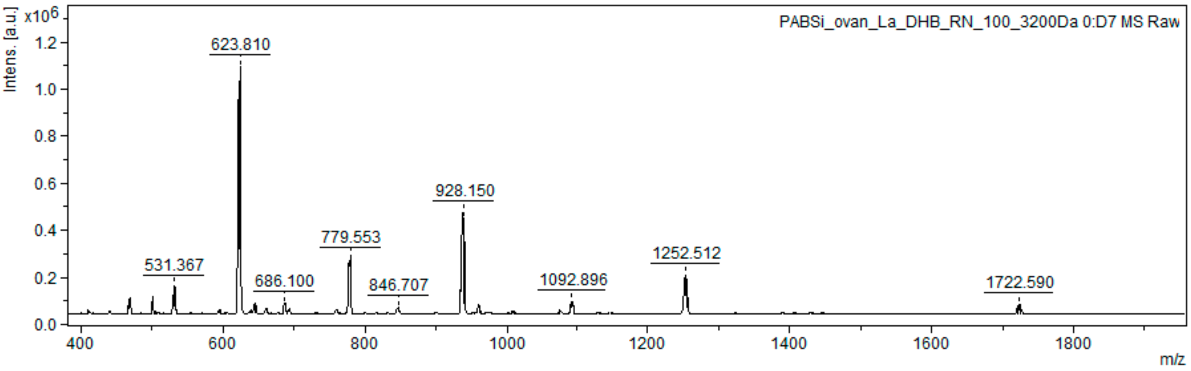

Figure S12. MALDI-TOF/ TOF-MS spectrum of [La**L2**$(NO_3)_2H_2O$]

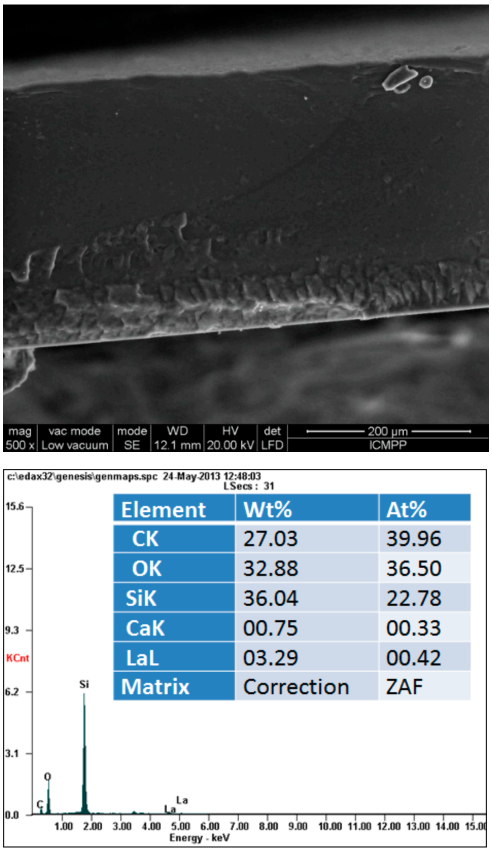

Element	Wt%	At%
CK	27.03	39.96
OK	32.88	36.50
SiK	36.04	22.78
CaK	00.75	00.33
LaL	03.29	00.42
Matrix	Correction	ZAF

Figure S13. SEM Cross-section in the LaSiOSi_PDMS films at different depths and EDX composition of the films

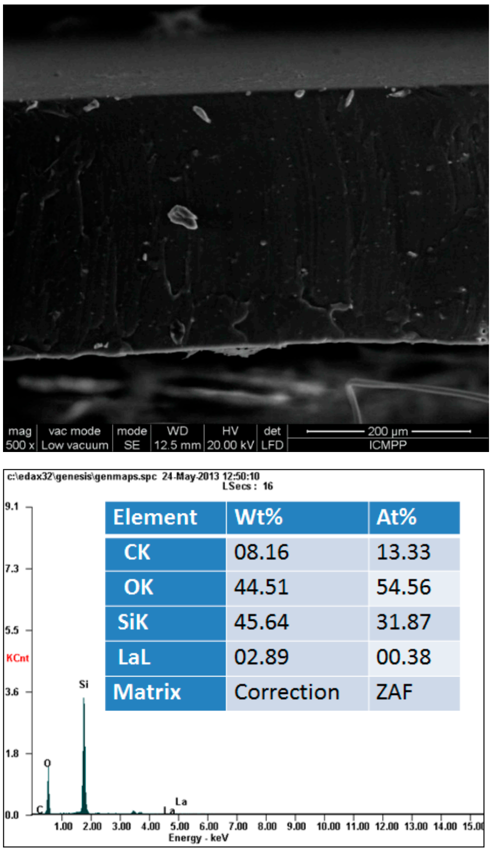

Element	Wt%	At%
CK	08.16	13.33
OK	44.51	54.56
SiK	45.64	31.87
LaL	02.89	00.38
Matrix	Correction	ZAF

Figure S14. SEM Cross-section in the LaSi_PDMS films at different depths and EDX composition of the films

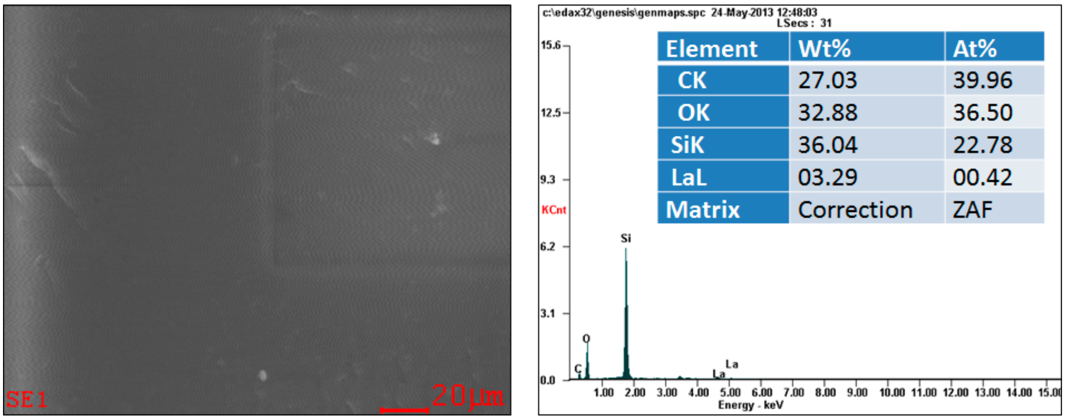

Element	Wt%	At%
CK	27.03	39.96
OK	32.88	36.50
SiK	36.04	22.78
LaL	03.29	00.42
Matrix	Correction	ZAF

Figure S15. SEM image of the surface of LaSi_PDMS film and the EDX composition in the selected region

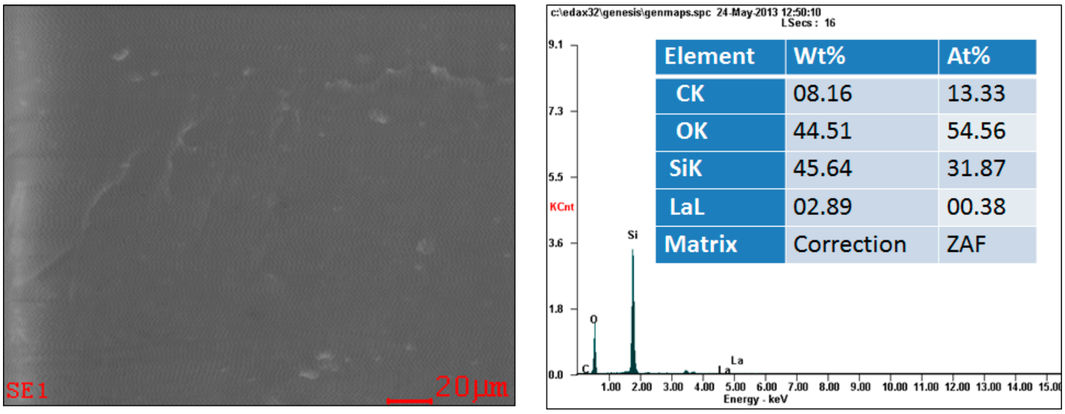

Figure S16. SEM image of the surface of LaSiOSi_PDMS film and the EDX composition in the selected region

S3. Thermal analysis of photoaged La(III) complexes – PDMS composites

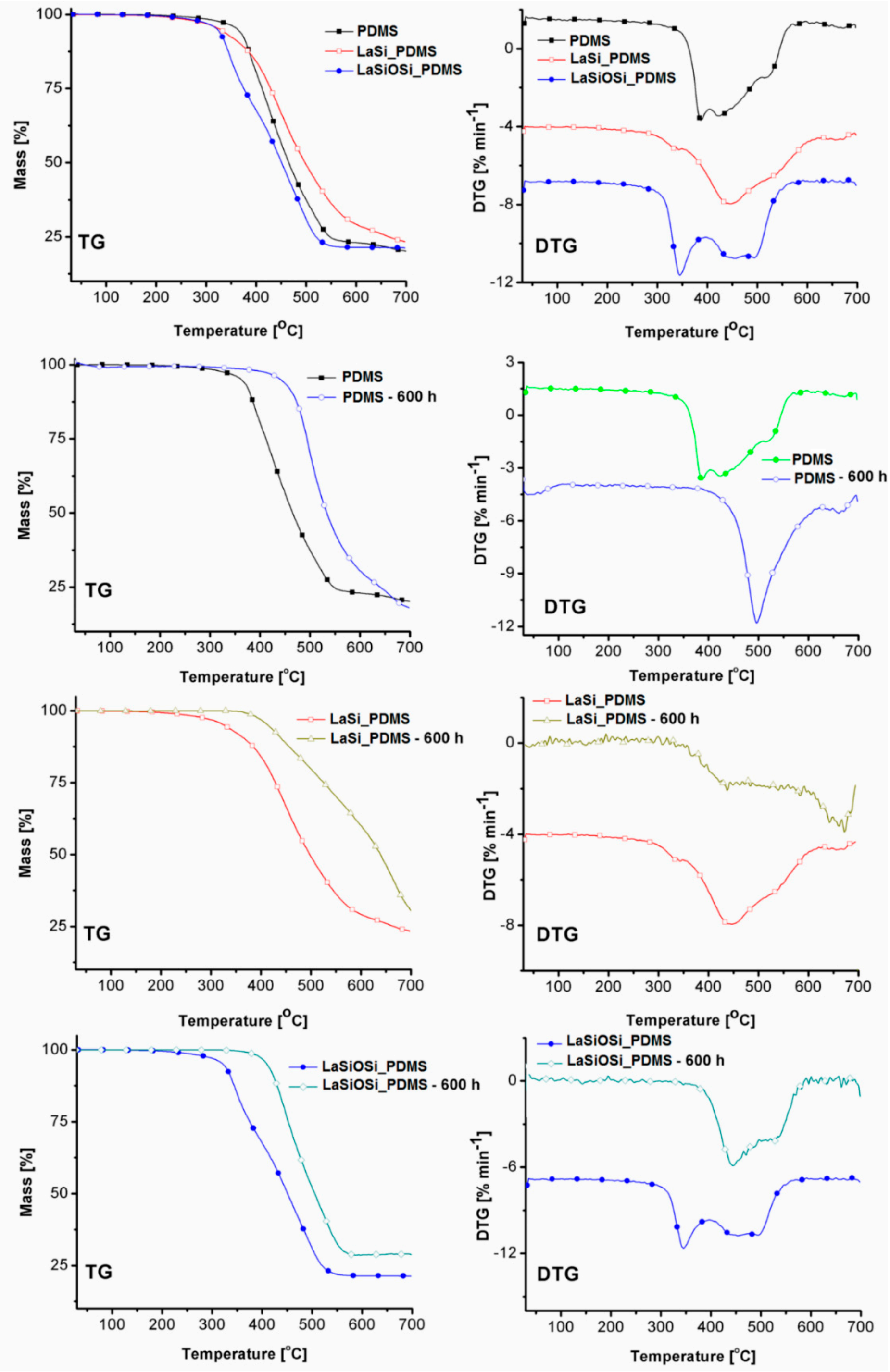

Figure S17. TG and DTG curves of initial samples and photo–aged for 600 hours.

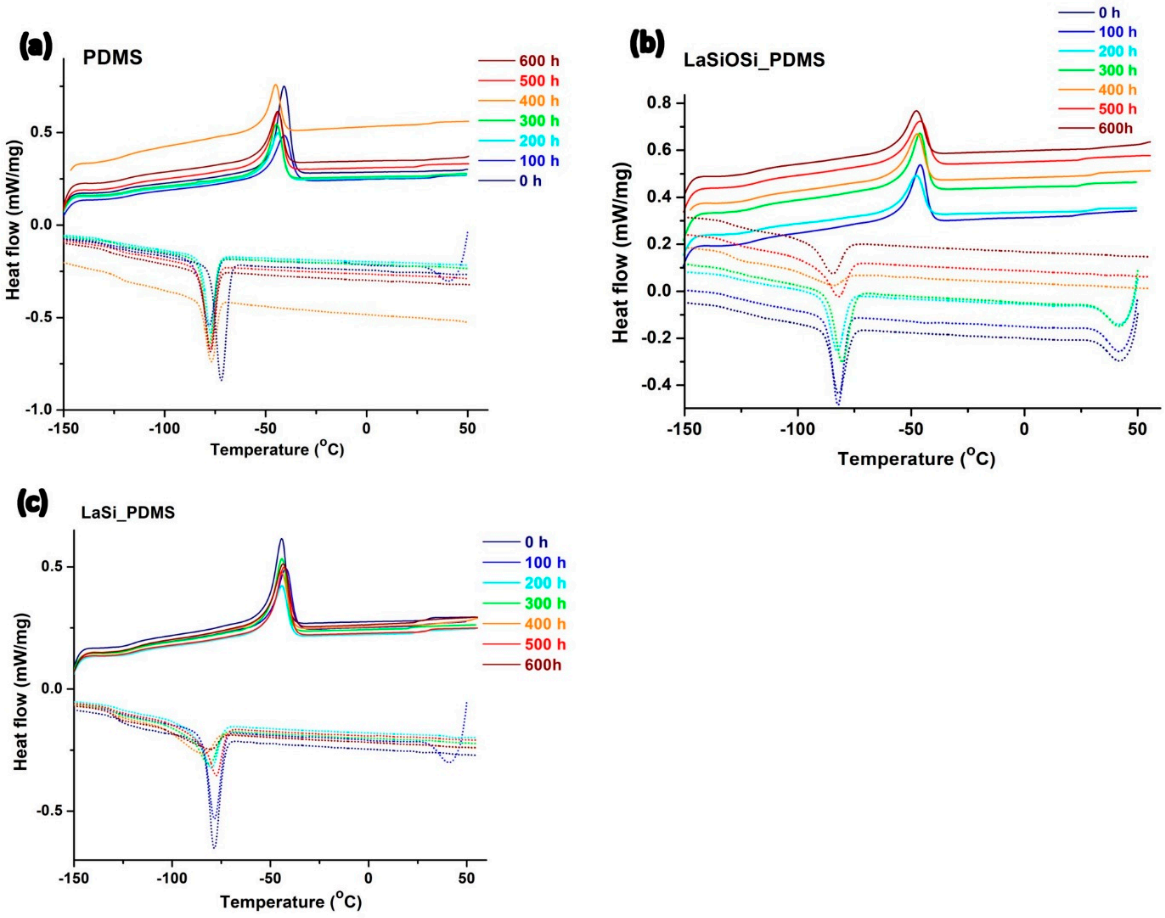

Figure S18. DSC thermograms of photoaged films: (a) native PDMSD; (b) LaSiOSi_PDMS; (c) LaSi_PDMS.

Table S1. Data extracted from the DSC curves of the studied sample.

Time (h)	Sample	T_{g1} (°C)	T_{m1} (°C)	ΔH_{m1} (J g^{-1})	T_{g2} (°C)	T_{m2} (°C)	ΔH_{m2} (J g^{-1})	T_{cr} (°C)	ΔH_{cr} (J g^{-1})	χ_c (%)
	M	–	–	21.66	–	–	21.68	–	–	35
0		123	41		122	41		72	23.99	
	F I	–	–	16.58	–	–	16.27	–	–	26
		120	44		120	45		79	16.54	
	F II	–	–	11.48	–	–	11.07	–	–	18
		121	48		120	48		82	11.73	
	M	–	–	13.14	–	–	13.95	–	–	23
100		118	41		118	41		78	16.46	
	F I	–	–	13.78	–	–	13.71	–	–	22
		120	42		119	42		79	14.28	
	F II	–	–	12.62	–	–	12.22	–	–	20
		120	46		121	46		81	13.34	
	M	–	–	12.2	–	–	12.15	–	–	20
200		119	44		120	44		77	14.19	
	F I	–	–	11.23	–	–	11.33	–	–9.82	18
		121	44		121	44		80		
	F II	–	–	9.428	–	–	9.469	–	–	15
		120	48		120	48		83	9.897	
	M	–	–	13.75	–	–	13.71	–	–	22
300		121	45		122	45		78	15.48	
	F I	–	–	14.83	–	–	14.5	–	–	23
		123	44		123	44		81	8.067	
	F II	–	–	12.32	–	–	12.35	–	–	20
		120	46		120	46		81	13.41	
	M	–	–	11.54	–	–	11.38	–	–	18
400		120	45		120	45		77	12.07	
	F I	–	–	13.32	–	–	13.44	–	–	22
		121	44		121	45		78	8.008	
	F II	–	–	11.21	–	–	11.04	–	–4.36	18
		122	47		123	48		84		
	M	–	–	14.96	–	–	14.37	–	–	23
500		119	44		119	44		78	16.36	
	F I	–	–	14.42	–	–	14.64	–	–	24
		121	43		121	43		84	11.86	
	F II	–	–	11.68	–	–	11.21	–	–	18
		120	46		122	46		82	9.097	
	M	–	–	13.53	–	–	13.53	–	–	22
600		121	44		121	44		77	15.87	
	F I	–	–	14.64	–	–	14.65	–	–	24
		121	43		122	44		80	6.006	
	F II	–	–	10.66	–	–	10.39	–	–	17
		122	48		122	48		85	8.067	

Short notations: M = PDMS, FI = LaSiOSi_PDMS; FII = LaSi_PDMS
T_{g1} – glass transition temperature corresponding to the first heating run;
T_{g2} – glass transition temperature corresponding to the second heating run;
T_{m1} – melting temperature corresponding to the first heating run;
T_{m2} – melting temperature corresponding to the second heating run;
ΔH_{m1} – enthalpy of the melting profile corresponding to the first heating run;
ΔH_{m2} – enthalpy of the melting profile corresponding to the second heating run;
T_{cr} – crystallization enthalpy;
ΔH_{cr} – crystallization enthalpy;
χ_c – degree of cristalnity calculated with the equation $(\Delta H t_2/\Delta H_{literature})\times 100$, where $\Delta H_{literature} = 61.3$ J g^{-1} .

S4. The complete series of the ATR-FTIR and fluorescence spectra of photoaged PDMS-based composites

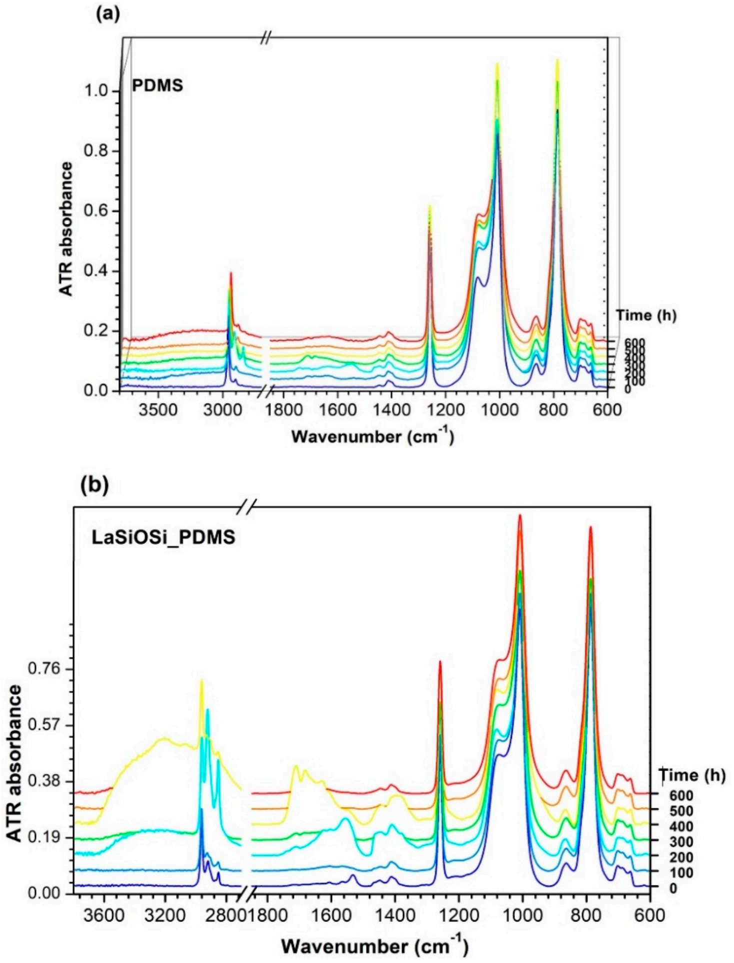

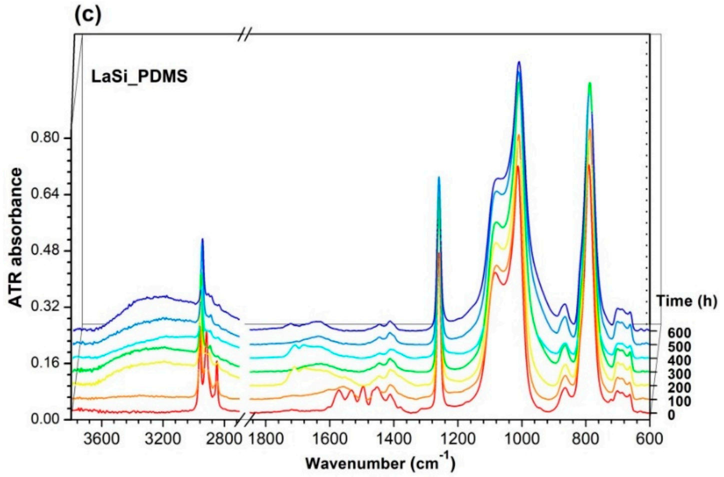

Figure S19. Evolution of infrared spectra during photoaging for: (a) neat PDMS (b) LaSiOSi_PDMS; (c) LaSi_PDMS.

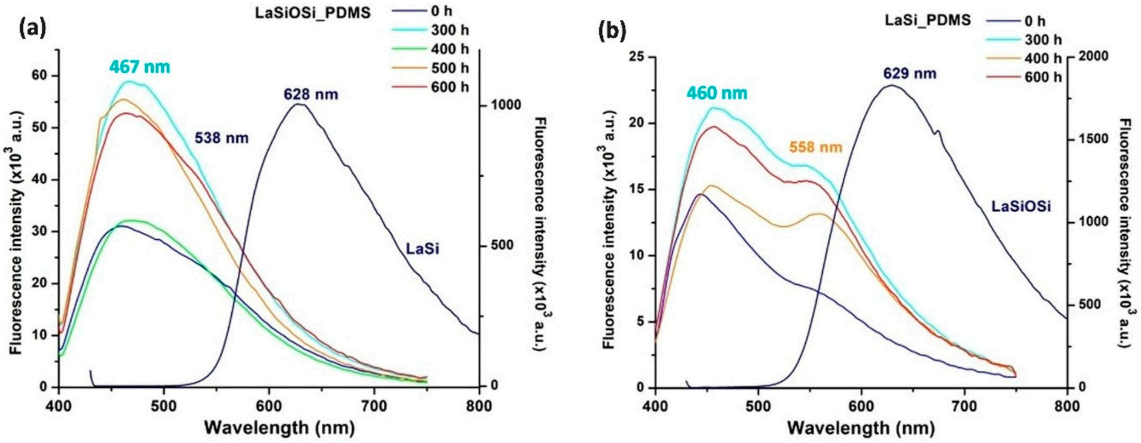

Figure S20. Effects of accelerated UV-aging on the fluorescence of LaSi/LaSiOSi_PDMS films compared to the powder form of pure complexes (dark blue line): (**a**) LaSiOSi_PDMS; (**b**) LaSi_PDMS.
